# Outlier analyses of the Protein Data Bank archive using a probability-density-ranking approach

**DOI:** 10.1038/sdata.2018.293

**Published:** 2018-12-11

**Authors:** Chenghua Shao, Zonghong Liu, Huanwang Yang, Sijian Wang, Stephen K. Burley

**Affiliations:** 1 RCSB Protein Data Bank, Rutgers, The State University of New Jersey, Piscataway, NJ 08854, USA; 2 Institute for Quantitative Biomedicine, Rutgers, The State University of New Jersey, Piscataway, NJ 08854, USA; 3 Department of Statistics and Biostatistics, Rutgers, The State University of New Jersey, New Brunswick, NJ, 08903, USA; 4 Rutgers Cancer Institute of New Jersey, Rutgers, The State University of New Jersey, New Brunswick, NJ, 08903, USA; 5 RCSB Protein Data Bank, San Diego Supercomputer Center and Skaggs School of Pharmacy and Pharmaceutical Sciences, University of California San Diego, La Jolla, CA 92093, USA

**Keywords:** Protein databases, Statistical methods, X-ray crystallography, Databases, Data processing

## Abstract

Outlier analyses are central to scientific data assessments. Conventional outlier identification methods do not work effectively for Protein Data Bank (PDB) data, which are characterized by heavy skewness and the presence of bounds and/or long tails. We have developed a data-driven nonparametric method to identify outliers in PDB data based on kernel probability density estimation. Unlike conventional outlier analyses based on location and scale, Probability Density Ranking can be used for robust assessments of distance from other observations. Analyzing PDB data from the vantage points of probability and frequency enables proper outlier identification, which is important for quality control during deposition-validation-biocuration of new three-dimensional structure data. Ranking of Probability Density also permits use of Most Probable Range as a robust measure of data dispersion that is more compact than Interquartile Range. The Probability-Density-Ranking approach can be employed to analyze outliers and data-spread on any large data set with continuous distribution.

## Introduction

The Protein Data Bank (PDB) supports secure storage and dissemination of three-dimensional (3D) structures of large biological molecules (proteins, DNA, and RNA)^1,2^. Founded in 1971 as the first open-access digital data resource in biology, the PDB has developed into the single global archive of >140,000 3D structures deposited by researchers worldwide, using experimental methods including Macromolecular Crystallography (MX), Nuclear Magnetic Resonance Spectroscopy (NMR), and Cryo-Electron Microscopy (3DEM). Many PDB structures represent groundbreaking scientific discoveries, garnering numerous Nobel Prizes, including five Chemistry awards in the 21^st^ century^3–11^. Since 2003, the PDB archive has been managed by the Worldwide Protein Data Bank partnership (wwPDB, pdb.org)^2^. Members of the wwPDB include the US RCSB Protein Data Bank^1,12^, Protein Data Bank in Europe^13^, Protein Data Bank Japan^14^, and BioMagResBank^15^. Over the past five decades, PDB data have enabled scientific breakthroughs in fundamental biology, biomedicine, and energy research^16^.

Archived data for each PDB structure, designated with a unique 4-character identifier (e.g., 1vol), include atomic coordinates, experimental data, and supporting metadata. The structure is a collection of the Cartesian coordinates of the atoms of the biomolecule in 3D space, whereas the experimental data and supporting metadata varies depending on the experimental method used to determine the 3D structure (MX: ~90%; NMR: ~9%; 3DEM: ~1%). Supporting metadata include the origin and biological characteristics of the macromolecular sample, experimental procedures, experimental data quality indicators, 3D structure quality indicators, and metrics used to assess consistency between atomic coordinates and experimental data. Over the past two decades, the US RCSB Protein Data Bank (RCSB PDB) has been analyzing trends for >130 PDB data items and using the results to guide PDB data deposition-validation-biocuration^17^ and enable maintenance of the PDB archive^18^. Our overarching goal is to ensure Findability-Accessibility-Interoperability-Reusability of PDB data^19^.

Since 2000, 3D structure data have been contributed to the PDB by >30,000 depositors worldwide. During processing (deposition-validation-biocuration) of incoming data^20–22^, it is not uncommon for wwPDB Biocurators to detect unusual values that represent either data entry mistakes or possibly scientific breakthroughs^23^, with the former being more common than the latter. Of particular concern during biocuration is question “How outlying is a particular datum?” A robust answer to this question is essential in any decision to initiate comprehensive review of data quality. It would, therefore, be highly advantageous to have a computationally simple, yet generally applicable, process with which to identify outliers across the entire corpus of PDB data with a minimum of false positives and false negatives.

An outlier is “an observation which deviates so much from other observations as to arouse suspicions that it was generated by a different mechanism”^24^. Commonly-used methods for outlier identification calculate the deviation from location and scale parameters to reveal the gap (or distance) between “outlying” and “inlying” observations^24,25^. *Z* score is a measure of the number of standard deviations from the mean value for Normal (Gaussian) distribution, where *|Z|* = 1.96 corresponds to 5% outliers and *|Z|* = 2.58 corresponds to 1% outliers. Employing more robust measures of location and dispersion, Tukey’s fences^26^ uses the Interquartile Range (IQR) to identify outliers as data falling below *Q1-k × IQR* or above *Q3 + k × IQR*, where *Q1* and *Q3* are 1^st^ and 3^rd^ quartiles, respectively, and *k* = 1 and *k* = 1.5 correspond to 5% and 1% outliers, respectively, for a Normal distribution. An alternative nonparametric method counts the number of Median Absolute Deviations (MAD)^27^ from the median. The above methods share the same concept of applying *n* standard deviations (*n*-deviation) symmetrically from the expected value, although the approaches to calculating the expected value and standard deviation are different. Finally, the simple-percent-cut method marks small percentiles such as 0.5 and 2.5% as outlier boundaries at both ends of the data distribution, but by using location parameters only this method is perhaps the least preferred because the distribution of the data *per se* is not considered. For example, the simple-percent-cut method fails on the distribution with data concentrated at the lower/upper bound, and on the multimodal distribution with outliers between modes.

Although these methods have been widely used to identify outliers of molecular geometry^28–32^, they do not work effectively for PDB data, which are usually characterized by heavy skewness, bounds of natural limit, or long tails. The skewness and long tails reflect the complexity of the structural data and metadata in the PDB archive. *Z* score or other parametric methods assume certain data distributions that are inconsistent with most PDB data. Tukey’s fences, MAD-based methods, and the simple-percent-cut assume outliers are roughly symmetric at both ends, but many PDB data distributions have highly asymmetric tails. Moreover, none of these methods can be properly applied to bounded data. An explicit probability-based approach would appear better suited to the diversity of data distributions represented among PDB data.

Herein, we demonstrate that effective outlier identification for PDB data can be accomplished by ranking the probability density for each datum. We employ kernel density estimation to estimate the probability density *f*(*x*), where *x* is the variable symbol of a PDB data item or parameter as described above. According to Silverman^33^, using a kernel estimator *K(u)* that satisfies $\int_{-\infty }^{\infty }K\left(u\right)du=1$ allows estimation of *f*(*x*) as follows:

(1)fˆ(x)=1nh∑i=1nK(x−Xih)

where *n* is the number of observations and *h* is bandwidth or window width used for kernel estimation^33^.

We have used the Gaussian kernel throughout this work, $K\left(u\right)=\frac{1}{\sqrt{2\pi }}\exp\left(-\frac{u^{2}}{2}\right)$. Overall errors of the probability density estimation can be described by Mean Integrated Square Error: $MISE\left(\hat{f}\right)=E\int^{\;}{\left(\hat{f}\left(x\right)-f\left(x\right)\right)}^{2}dx$. Optimal bandwidth *h* can be found by minimizing the *MISE*^33^. A commonly used approach is to use reference of standard Normal probability density, which gives $h_{opt}=1.06\;\sigma \;n^{-1/5}$. A more robust version replaces *σ* with *IQR/1.34*, yielding the following:

(2)hopt=0.79IQRn−1/5

Simulation studies indicate that the *IQR* version above is a better approximation to the asymptotically optimal bandwidth for data with skewness and kurtosis^33^.

We then applied the Probability-Density-Ranking method to 22 PDB data sets selected on the basis of their significance in the field of structural biology. Although the conventional *n*-deviation and simple-percent-cut approaches do not work properly on some data due to their skewness, bounds, or long tails, they are still used frequently, albeit incorrectly, in the field to detect ‘outliers’. Therefore, by comparing the results from Probability-Density-Ranking to those from other outlier identification methods, we demonstrate that our new method is more effective in finding the least probable data of a variety of different types of distribution. This approach can also be applied on any scientific data with continuous distributions and sufficient observations to generate reliable probability density estimates.

## Results

### Outlier Identification Based on Probability Density Ranking

**Figure 1:**
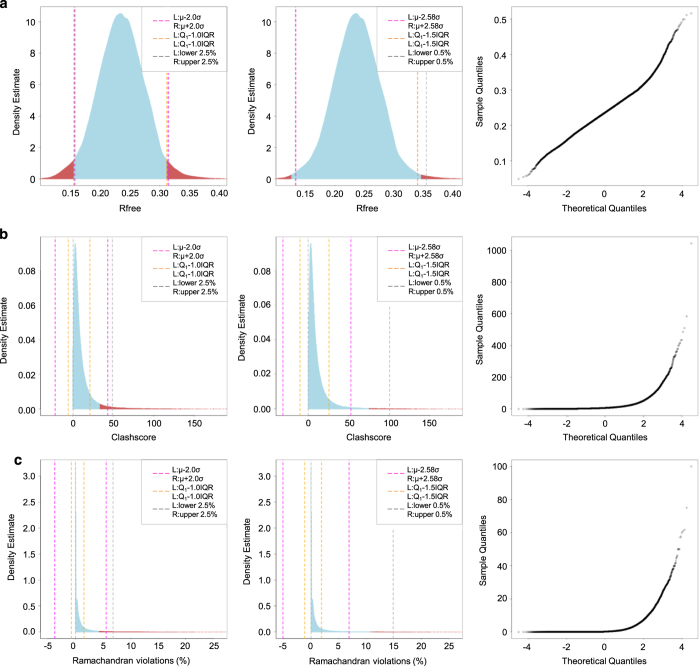
Data distribution and Probability-Density-Ranking (PDR) outliers of PDB structure quality indicators. (**a**) Rfree distribution in the first row, (**b**) Clashscore distribution in the second row, and (**c**) Percent of Ramachandran violations in the third row show probability density estimate with 5% and 1% PDR outliers, as well as Normal Q-Q plot, from left to right. The 5% (left) and 1% (middle) PDR outlier regions are colored in red, and the rest of the data range in light blue. As comparisons, The outlier boundaries identified by conventional methods are marked by vertical dashed lines of the following color designation: magenta for *Z* score, orange for Tukey’s fences, and grey for simple-percent-cut.

Probability density distribution of three primary PDB structure quality indicators^21^ are displayed in Fig. 1. Rfree in Fig. 1a is a quality metric specific to PDB structures determined by MX, measuring consistency between deposited atomic coordinates and associated experimental diffraction data^34^. Smaller values of Rfree indicate superior consistency, and the value is bounded between the natural limits of 0 and 1. Probability density of Rfree for 123,849 PDB MX structures (Data Citation 1) was estimated using Equation (1), with Gaussian kernel and bandwidth values from Equation (2). The probability density estimate, as shown in Fig. 1a left and middle panels, displays a bell-shape resembling a Normal distribution with a slightly heavy tail at the upper end. The Normal Q-Q plot (Fig. 1a right) also exhibits a near-straight diagonal line except at the extreme tail regions.

The probability density estimate for each PDB structure was then ranked, and those falling below a set threshold were identified as Probability-Density-Ranking (PDR) outliers. Rfree values with the lowest 5% or 1% probability density estimate are denoted in red in Fig. 1a left and middle panels, respectively. PDR outliers were then compared to outliers determined by the *n*-deviation approaches including *Z* score and Tukey’s fences, and by the method of simple-percent-cut, with boundaries marked by dashed lines colored magenta, orange, and grey, respectively. 5% PDR outliers are consistent with those from other methods, as demonstrated by the overlapping outlier boundaries and the exact values in Supplementary Table S1. Our results document that for the majority data at 0.1–0.4 range with near-Normal distribution, the PDR and conventional methods set consistent outliers boundaries. On the other hand, at the extreme tail regions where Normality is broken, the outlier consistency is no longer held, as demonstrated by that 1% PDR outliers differing from those identified with the other methods duo to slight skewness of 0.17 and kurtosis of 3.96 particularly within the tail regions (Fig. 1a and Supplementary Table S1).

Clashscore is a scaled measure of the number of energetically unfavorable non-bonded, atom-atom clashes within a 3D structure of a biological macromolecule, calculated using Molprobity^35^ (Data Citation 1). Clashscores have a natural lower limit of zero (i.e., no atom-atom clashes detected). Higher values indicate more interatomic clashes. Hence, the distribution of Clashscore is not Normal. For this type of heavily right-skewed distribution with a very long right-sided tail, the PDR method and other conventional methods are expected to have different outliers identified. Outcomes of 5% PDR outlier estimation were compared to corresponding outcomes from other methods (Fig. 1b left). Both Z score and Tukey’s fences methods fail to discriminate between the bulk of the data and the desired 5% of least probable outliers for Clashscore. At the lower end of the distribution, negative outlier boundaries corresponding to *μ-2σ* (*Z = *−2, magenta line) and *Q*_*1*_*− 1.0 × IQR* (orange line) are meaningless, because they extend beyond the natural limit. At the upper end of the distribution, *μ + 2σ* identifies 3.2% of data as outliers and *Q*_*3*_* + 1.0 × IQR* identifies 11.2% of data as outliers. The simple-percent-cut (5%) of Clashscore (grey lines) sets the lower outlier boundary at 0.45 and upper boundary at 49.12 (Supplementary Table S1). But, visual inspection of the data shows that Clashscores falling between 0 and 0.45 are not rare events. In fact, 1.4% PDB structures have zero non-bonded inter-atomic clashes, the hallmark of a very well determined 3D structure. Furthermore, the simple-percent-cut method assumes that both tails in the data distribution are equally likely, yet the probability density estimate at 0.45 is 42 times higher than the corresponding value at 49.12. The violation of symmetry assumption leads to an inadequate performance of the simple-percent-cut method.

Figure 1b middle panel compares 1% PDR outliers to the outcome of the other three methods. Since *σ* and *IQR* are dominated by data-rich regions in the distribution, they are less sensitive to the presence of a long right-sided tail. Boundaries at *μ + 2.58σ* and *Q*_*3*_* + 1.5 × IQR* identify 2.2 and 8.2% data, respectively, on the upper end of the distribution as outliers, both far exceeding the desired 1% least probable outliers. For the simple-percent-cut (1%) method, the lower outlier boundary is meaningless as explained above.

Ramachandran violations indicate that an amino acid within a polypeptide chain has spatially-incompatible backbone dihedral angles in the vicinity of the peptide bonds connecting successive amino acids^36^. Percent Ramachandran violations are also calculated by Molprobity^35^ (Data Citation 1), with natural limits between 0 and 1. Figure 1c shows a skewed distribution of the percent of Ramachandran violations with a significant right-sided tail. 43.9% of the PDB structures have no Ramachandran violations, thus the natural lower limit of 0 is actually the mode, which renders lower boundaries identified by σ, *IQR*, or simple-percent-cut methods meaningless. Similarly, at the upper end of the distribution, none of the three conventional methods yield meaningful outlier boundaries. In contrast, the PDR method identifies Ramachandran violations exceeding 0.043 as outliers, corresponding to 5% of the least probable data.

As demonstrated in Supplementary Table S1 and Supplementary Figure S1, typical PDB metadata distributions display features similar to those seen in Fig. 1b,c -- skewness, long tails, and natural limits. Some data also exhibit multimodal and delta-function-like distributions. Even for these more complicated data distributions, the PDR method is applicable and can be used to identify outliers with more confidence than conventional methods.

### PDR Outliers Used for PDB Data Curation

**Figure 2:**
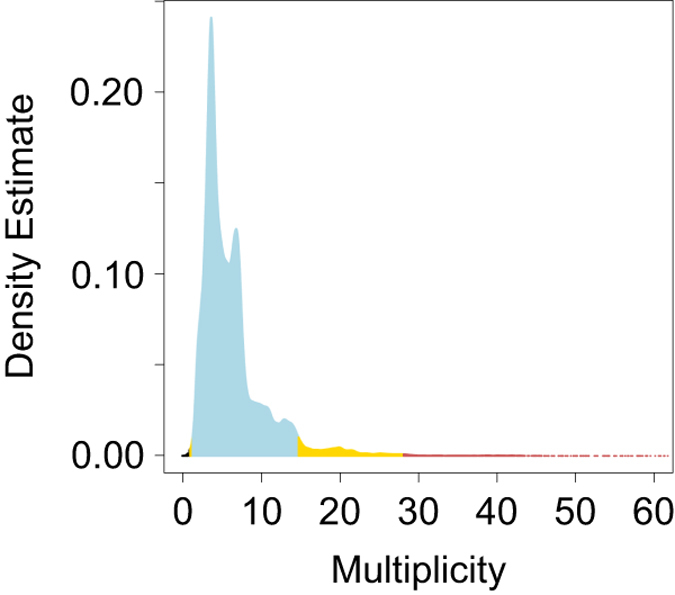
Data distribution and PDR outliers of MX Data Multiplicity. 1% PDR outlier region is colored in redred, 2–5% PDR outlier region in yellow, and observations beyond natural limit in black. The long right-sided tail is truncated at 60 for the sake of clarity.

As demonstrated, PDR method is equally affective as the *n*-deviation and simple-percent-cut methods for normally distributed data, and more effective in identifying the exact percent of least probable data in skewed and long-tailed distributions commonly observed in PDB. Therefore, the PDR method is of practical use as an uniform outlier identification process for data quality control during deposition-validation-biocuration of PDB structures. For example, wwPDB Biocurators review Data Multiplicity for MX structures, also known as data redundancy, a measure of the average number of measurements of each symmetrically unique crystallographic data reflection (Data Citation 1). Figure 2 shows the distribution of Multiplicity of the current PDB archive. 34 PDB structures have apparent Data Multiplicity below the natural lower limit of 1.0, colored black in Fig. 2. As outlier examples, two PDB structures (2jlo and 2jl3)^37^ both had the lowest Multiplicity of 0.01, but these structures were subsequently obsoleted and Data Depositors provided new data with correct values of 2.9 (2xxg) and 3.2 (2xxf)^37^, respectively. PDB structure 3mby has the third lowest apparent Multiplicity value of 0.035 that proved to be a data entry error, and true value of 3.5 was reported in the associated publication^38^. These and other entries with data redundancy less than 1.0 were excluded from our analyses, and will be corrected during the course of routine archive remediation activities.

Given the heavy skewness and long right-sided tail of the Data Multiplicity distribution, the PDR method was effective in identifying outliers. Lower outlier boundaries based on *Z* score and IQR are both negative, and, therefore, meaningless (Supplementary Table S1). Simple-percent-cut outlier identification failed because lower outlier boundaries for both 1% and 5% simple-percent-cuts encompassed a significant number of legitimately determined PDB structures dating from an era when redundant data collection was technically challenging. In contrast, the PDR method placed the 5% lower outlier boundary at 1.3, encompassing 221 structures (There is no PDR 1% lower outlier boundary in this case). Review of the these lower boundary 5% PDR outliers suggests that PDB Data Depositors made data entry errors in most of these cases, which could have been prevented at the time of data deposition.

Upper boundary 5% and 1% PDR outliers occur at 14.8 and 28.1, respectively. While unusual, neither of these findings are at odds with what is achievable with newer measurement techniques. For the earliest structures deposited to the PDB, diffraction Data Multiplicity was limited by deterioration of crystals during data measurement. The advent of Cryo-Crystallography^39^ and access to brighter X-ray sources have significantly increased the likelihood of collecting MX data with high Multiplicity. Indeed, the median value of Data Multiplicity grew from 3.0 in 1994 to 6.2 in 2017. The greatest impact on Data Multiplicity in the past few years came from introduction of X-ray Free Electron Lasers (XFEL) supporting Serial Femtosecond Crystallography (SFX)^40^ studies of multiple crystals, generating data of extremely high Multiplicity. Fully 81% of PDB structures from XFEL facilities fall within upper boundary 1% PDR outliers (Data Multiplicity > 28.1), including PDB structure 4zix^41^ with Multiplicity of 26,558. It is clear, therefore, interpretation of the PDR analyses requires knowledge of the experimental context. Analyzing data on the basis of data collection protocol (SFX subset *versus* non-SFX) will be necessary in future to distinguish Data Depositor errors from legitimately high Data Multiplicity values arising from SFX. At present, upper boundary 5% PDR outliers occur at 27.6 and 560.0 for non-SFX structures and SFX structures (244 total), respectively.

### Local Data Clusters

**Figure 3:**
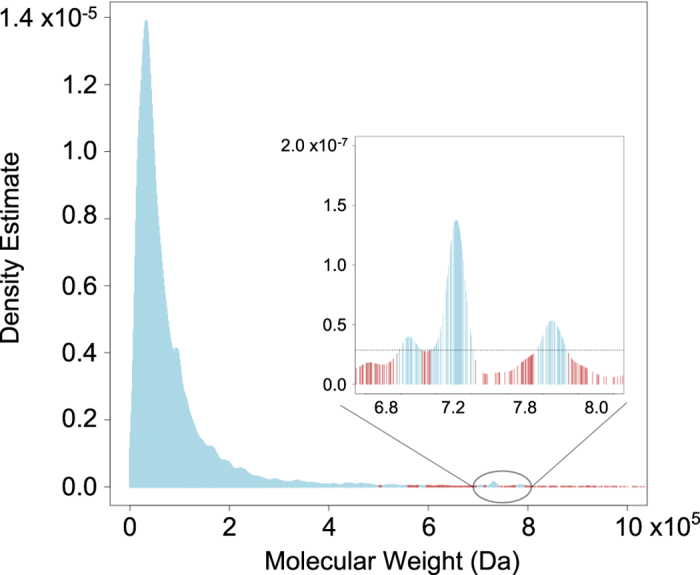
Data distribution of Molecular Weight and 1% PDR outliers. The cumulated Molecular Weight in the Asymmetric Unit of each MX structure model is used for calculation. Inset shows the region between 680000 and 800000 with local data clusters.

The *n*-deviation and simple-percent-cut methods simply set lower and upper boundaries without consideration of the local features around the boundaries, under the assumption that the probability of data presence beyond the boundaries diminishes continuously. This assumption may not hold if there are local maxima beyond the boundaries, whereas PDR outlier analyses, which avoid such assumption, make it possible to discover data clusters falling within the tails of distributions representing interesting subsets of PDB structures. Figure 3 illustrates the distribution of Molecular Weight (MW) in the Asymmetric Unit of PDB MX structures (Data Citation 1). PDB structures span an extremely wide range of MW, from the lowest value of 468 Dalton (Da) for a small D-peptide (6 anm)^42^ to the current maximum value of 97,730,800 Da for an Adenovirus capsid (4cwu)^43^. Like 4cwu, many of the PDR outliers greater than 1,000,000 are virus structures or other large macromolecular assemblies falling into particular subsets. (N.B.: Figure 3 was truncated at an upper limit of 1,000,000 Da for the sake of clarity).

Figure 3 inset shows that the probability density estimate fluctuates within the tail region, resolving into three distinct clusters (light blue) for PDB structures with probability greater than the 1% PDR outlier threshold. Examination of these clusters reveal that they are structures of the same or similar macromolecules that were the foci intensive investigation. The larger peak at MW = 717341–740854 Da encompasses ~200 similar PDB structures of a large molecular complex known as the proteasome (a protein degradation machine). Approximately half of the 62 PDB structures with MW = 699159–711565 Da are also proteasomes, albeit with different amino acid sequences and MWs. Nearly 80% of the 100 PDB structures within MW = 777289–793443 Da, are structures of small ribosomal subunits. These findings underscore the fact that target selection in structural biology research is not a simple random process, instead researchers study particular macromolecular assemblies (such as proteasomes and ribosomes), because they are important actors in human health and disease and fundamental biological processes. The PDR method enabled identification of unusual clusters at the right-sided tail of the MW distributions.

### Matthews Coefficient and Most Probable Range

**Figure 4:**
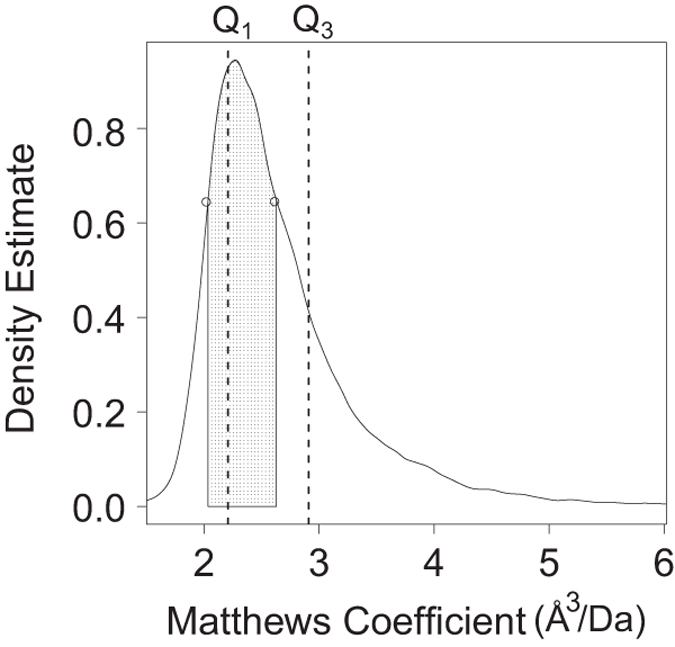
Data distribution of Matthews Coefficient and the Most Probable Range. 50% MPR is shown in shaded area. The median of probability density estimates (y-axis) for all observations is 0.648, the height of the two small circles that correspond the Matthews Coefficient values (x-axis) of 2.04 (left) and 2.63 (right), respectively. The MPR range between 2.04 and 2.63 contains 50% observations with probability density estimates higher than the median of 0.648, i.e. the 50% most probable observations. IQR between the perpendicular dashed lines at *Q*_*1*_ (left) and *Q*_*3*_ (right) is shown for reference.

A useful computed quantity, known as the Matthews coefficient with units of Å^3^/Da, was introduced in 1968 to assess molecular packing density within protein crystals^44^. In 1968, mean and median values for Matthews coefficients were 2.37 and 2.61, respectively, based on 116 MX structures^44^. Since that time such calculations have been updated several times as more and more structures entered the PDB^45–48^. In 2014, analysis of 60,218 PDB MX structures yielded mean and median values of 2.67 and 2.49, respectively^48^. The current distribution of Matthews coefficients calculated in 2018 for 128,668 PDB MX structures (Data Citation 1) is illustrated in Fig. 4. Both mean (2.67) and median (2.5) values are essentially unchanged from 2014, with mode shifted slightly to a value of 2.27 from 2.32 in 2014. Upper and lower 5% PDR outlier boundaries are 4.1 and 1.72, respectively. Upper and lower 1% PDR outlier boundaries are 5.32 and 1.48, respectively, both representing extreme values that should be manually reviewed during PDB structure biocuration.

For the benefit of macromolecular crystallographers, we also define the Most Probable Range (MPR) of Matthews coefficient as the 50% of observations with the highest probability density estimates. The MPR range of 2.04-2.63 (Fig. 4) encompasses the 50% most probable Matthews coefficients in the PDB archive today. Although IQR is commonly used as the 50% data range of general data distribution, Fig. 4 demonstrates that IQR does not represent the range of the most probable Matthews coefficients, because of the markedly asymmetry of the data distribution. If one assumes that the probability density estimation has the same order statistic as that of the true probability, MPR has the highest breakdown point of 50%, making it a more robust range measure than IQR. As a practical guide, the probability of a particular Mathews coefficient can help structural biologists to determine the most likely number of molecular instances comprising the asymmetric unit^45^, and MPR is an accurate quantitative measure with which to distinguish more likely from less likely choices of asymmetric unit contents during the early stages of a new structure determination. MPR of other PDB data are also calculated (Supplementary Table S2).

### Conditional Data Distribution

Since the PDB archive houses a diverse collection of 3D structures of biological macromolecules determined using different experimental methods, it is sometimes useful to analyze data distributions for similar molecules coming from the same type of experiments. Figure 2 documented that the observed distribution of certain PDB data may be confounded by inclusion of data from different MX data collection methods. In this situation, conditional data distributions can be used to divide PDB data into homogenous subsets, making it easier for wwPDB Biocurators to identify extraordinary PDR outliers that are frequently the product of human error. Conditional data distributions should be used for other types of PDB data as well. For example, the datum known as B factor, also called “temperature factor” or “Debye Waller factor”, is a measure of positional uncertainty of an atom or a group of atoms^31^. B factor distributions have a natural lower limit of zero with higher values indicating greater levels of uncertainty. Both very high and very low B factors can be result from errors in MX structure determination^49^. We also know that higher B factors are frequently associated with a paucity of diffraction data, thus any analysis of outliers should be made with respect to diffraction data resolution limit (lower values indicate more data)^49^.

**Figure 5:**
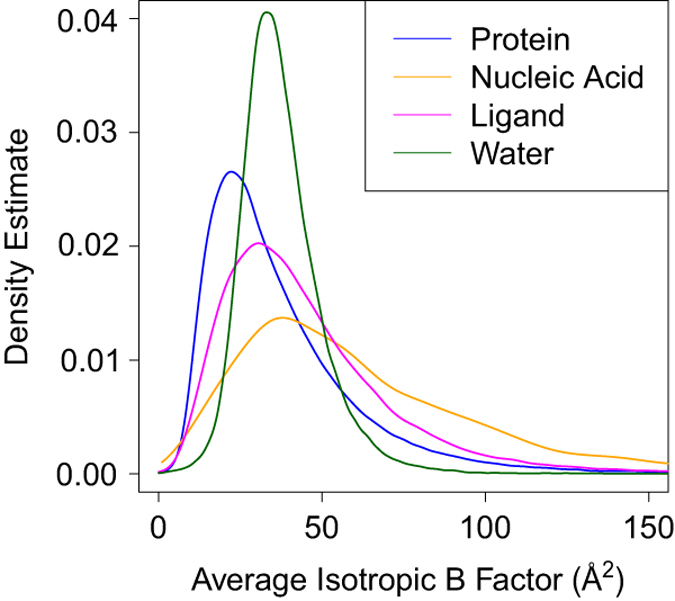
Conditional data distribution of average isotropic B factors. Distributions of different types of chemical components are illustrated by lines in different color: blue for protein, orange for nucleic acid, magenta for ligand, and green for water.

B factor data distributions can also be influenced by the type of biological molecules. Figure 5 displays the distribution of averaged B factors as a function of molecular type. The average B factor was obtained by averaging each atom record of a particular type of component in a PDB model (Data Citation 1). These results reveal that the atoms comprising the proteins have lower average B factors (i.e., less positional uncertainty) than the atoms comprising nucleic acids or ligands, indicating greater spatial uncertainty for such components in general. The distribution of B factors for partially-ordered water molecules present on the surface of biological macromolecules is quasi-symmetric with a mode exceeding corresponding values for protein and ligand atoms. Not surprisingly, upper and lower PDR outlier boundaries differ for each type of of MX molecular samples (Supplementary Table S1). The conditional distribution based on different type of experiments are also demonstrated in Supplementary Figure S2.

## Discussion

Many of the metadata items stored in the PDB archive do not exhibit distributions commonly presented in introductory statistics textbooks (e.g., Normal, Lognormal, and Gamma distributions). Instead, PDB data distributions are frequently bounded, skewed, and multimodal, with long left- or right-sided tails. We have presented illustrative case studies drawn directly from PDB data that demonstrate the power of the PDR nonparametric method for analyzing distributions and identifying outliers. The kernel density estimate represents a reliable means of assessing the probability of continuous PDB data distributions of large data.

We can also assess the variance of the probability density estimate under continuous assumption, by $var\left(\hat{f}\left(x\right)\right)=\frac{1}{nh}f\left(x\right)\int t^{2}K\left(t\right)dt+O\left(\frac{1}{nh}\right)$^50^. When *h*→0, *O*(*h*^4^) and $O\left(\frac{1}{nh}\right)$ are of smaller order than the corresponding leading terms, and the variance can be approximated by replacing *f*(*x*) with $\hat{f}\left(x\right)$. Variance estimates can be used to determine whether or not the probability density estimates of two observations are significantly different, and such calculations have been implemented (Data Citation 1).

Probability kernel density estimation relies on bandwidth selection. There are two types of bandwidths: fixed-length and adaptive bandwidth, and multiple calculation methods were developed for both. A special type of k-Nearest-Neighbor (kNN) kernel uses fixed number of data observations. By comparing results from each choice of kernel (Supplementary Table S3 and Figures S3 and S4), we determined that the bandwidth given by Equation (2) represents a reasonable initial choice, with most kernels yielding similar sets of PDR outliers. We also observed that larger bandwidth and 5% PDR outliers should be used if the goal is to have a crude range and outlier assessment, whereas smaller bandwidth and 1% PDR outliers should be used if one needs to study local distribution features.

Complementing PDR outliers, MPR measures the spread of the ‘central’ data-rich region. MPR has a breakdown point of 50%, much higher than the 25% breakdown point of IQR. For symmetric distributions, MPR converges to IQR, whereas for asymmetric single-peak distributions commonly observed in PDB, MPR is always smaller than IQR (Supplementary Tables S1 and S2). MPR of Matthews coefficient is 15% smaller than IQR, yet any randomly selected Matthew coefficient value has a 50% chance of falling into this compact range. Therefore, MPR represents the narrowest range to measure data aggregation. We can also extend MPR to cover other specified percentage of most probable data. 50–95% MPR of 22 PDB datasets are shown in Supplementary Table S2, which also demonstrates the 95% MPR complement the 5% PDR outliers.

For unimodal distributions that are monotonically increasing to the left of the mode and monotonically decreasing to the right of the mode, MPR is a single range of the shortest interval covering a specified fraction of data. For multi-modal distributions, such as Data Multiplicity (Fig. 2), the definition of MPR can be extended to the combination of intervals with the smallest total length covering certain fraction of data. For example, 65% MPR of Data Multiplicity includes two ranges of 2.6–5.8 and 6.0–7.3. The definition of MPR can be also applied to discrete distributions based on data frequency count, and further extended to multivariate distributions, which represent the smallest area or volume covering specified percent of the data. Although a similar concept has been used in structural biology to construct the 2-Dimensional Ramachandran plot^35,36,51^, the definition of MPR and the analysis of its properties will further its usage as a most robust range measure.

Based on our analyses of PDB data, we suggest the following approach for depositing-validating-biocurating data with continuous distributions that are coming into established repositories:

- Select bandwidth for probability density estimation (use a smaller bandwidth if the focus is on local features, and larger bandwidths for studying general distributions);
- Review 1% and 5% upper and lower boundary PDR outliers. If many of the apparent outliers represent a different mechanism of data generation, use conditional distribution to split data and conduct separate PDR outlier identifications in parallel;
- Investigate all apparent outliers to identify (and correct) data entry errors and outliers that may represent unusual/unexpected research findings meriting further examination.

This recommendation assumes that the majority of the data already in the data repository are of sufficient quality, and that such information will drive the process of identifying existing outliers (that should be remediated) and identifying erroneous outliers among incoming datasets (thereby preventing their incorporation into the data resource). Central to the success of any push to improve data quality, will be the level of information provided to Data Depositors when outliers are identified. We, therefore, further suggest that validation reports provided to Data Depositors be accompanied with information regarding 50%–95% MPR ranges of relevant data distributions, to enable understanding of particular data items.

## Methods

### Data Source and Data Process

Data were obtained from the open-access PDB archive (http://www.rcsb.org/) as of June 27, 2018. Data sets of >130 metadata items were extracted from PDBx/mmCIF model files and loaded into a MySQL Database (https://www.mysql.com/). Molprobity data were extracted from wwPDB validation report^21^ XML files located at ftp://ftp.wwpdb.org/pub/pdb/validation_reports/, with XML schema described in https://www.wwpdb.org/validation/schema/wwpdb_validation_v002.xsd. Database queries were used to generate customized data sets of the interest. A total of 22 such data sets were studied, including 12 displayed in Supplementary Figure S1. The following data sets are on MX structures only: Rfree, reflection data multiplicity, molecular weight in asymmetric unit, crystal Matthews coefficient, average B factor of protein atoms, average B factor of nucleic acid atoms, average B factor of ligand atoms, average B factor of water atoms, B factor estimated from Wilson plot, crystal solvent percentage, crystal mosaicity, Rfree minus Rwork, reflection high resolution limit, reflection data indexing chi-square, reflection data Intensity/Sigma, reflection data Rmerge, reflection data completeness, and percent RSRZ outliers. Molecular weight in asymmetric unit data is from an archive snapshot on March 14, 2018 and Noncrystallographic Symmetry correction was applied to construct complete asymmetric unit on ~260 PDB entries with minimal asymmetric unit in the model file.

All selected data were cleaned and formatted using Python (https://www.python.org/), and all PDB data with values falling within natural limits were included in our analyses. A very small number of data points with unrealistic values (i.e., those beyond natural limits, such as negative absolute temperature) were excluded from our analyses and are now undergoing remediation within the archive. All data sets were uploaded with this manuscript. Supplementary Table S1 enumerates the content and size of each data set. Size differences reflect (i) the characteristics of the data (e.g., there are significantly fewer nucleic acid structures than protein structures), and (ii) the fact that some items are not mandatory (e.g., It’s optional for author to provide B factor estimated by Wilson plot).

### Data Analyses

Computational analyses were carried out primarily with R (https://www.R-project.org/) in Linux parallel mode. Probability density for each data set was estimated using Equation (1) with a Gaussian kernel, and bandwidth determined by Equation (2). For comparison among different bandwidths (Supplementary Table S3), the kedd software package^52^ was used for all fixed-length bandwidth selection methods except for h.iqr that is in-house implementation based on Equation (2), and the default Gaussian type kernel was used for calculation from fixed-length bandwidths. The two adaptive kernel estimations were both in-house implementations: k-Nearest Neighbor (kNN) estimation was based on

(3)fˆ(x)=1ndk(x)∑i=1nK(x−Xidk(x))

with *d*_*k*_(*x*) being the *k*-th order nearest neighbor around x, and a bandwidth *k*=200 (2% sample size); Two-step variable kernel estimation was based on formula 2.31 in section 2.10.2, page 42 of the 1995 edition of “*Kernel Smoothing*” by Wand & Jones^53^, using Gaussian type kernel at variable bandwidths. The other names and references of kernel bandwidth selection in Supplementary Table S3 are of the following: h.amise, based on Asymptotic Mean Integrated Squared Error^54^; h.bcv, based on Biased Cross-Validation^55^; h.ccv, based on Complete Cross-Validation^56^; h.mcv, based on Modified Cross-Validation^57^; h.mlcv, based on Maximum-Likelihood Cross-Validation^58^; h.tcv, Trimmed Cross-Validation^59^; h.ucv, Unbiased (Least-Squares) Cross-Validation^60^. 10,000 simple random sample of the estimated B factor data set were used because some bandwidth calculation methods in kedd package need excessive memory if the full data set was used. Uniform kernel was also implemented as a comparison to Gaussian kernel, and the calculation based on Uniform kernel is in-house implementation.

Figures were drawn using R. For the sake of clarity, probability density plots were cut at 0.1% or 99.9% percentile at the very long tail at the lower or upper ends, unless otherwise indicated in the figure legend. R codes have been uploaded with this manuscript, together with the resulting data sets, bearing additional column to indicate whether the value in the row is 5% PDR or 1% PDR. Supplementary Tables S1 and S2 lists MPR and other statistics of all 22 data sets. “NA” is used in tables if there is no outlier at the specified end for a set threshold. For MPR calculation, if the left/right natural limit probability is greater than median probability, we used natural limit value as MPR left/right boundary. MPR was only calculated for single-peaked distribution with one range of MPR.

### Code and Data Availability

The data and R code to re-produce the results have been uploaded to FigShare (Data Citation 1) and GitHub: https://github.com/rcsb/PDB-Outlier-Analysis.git

## Additional information

**How to cite this article**: Shao, C. *et al*. Outlier analyses of the Protein Data Bank archive using a probability-density-ranking approach. *Sci. Data*. 5:180293 doi: 10.1038/sdata.2018.293 (2018).

**Publisher’s note**: Springer Nature remains neutral with regard to jurisdictional claims in published maps and institutional affiliations.

## Supplementary Material

Supplementary Information
