# Supplementary material for: Outlier analyses of the Protein Data Bank archive using a probability-density-ranking approach
Source: Sci Data. 2018 Dec 11;5:180293. doi: 10.1038/sdata.2018.293 (PMC6289109; doi:10.1038/sdata.2018.293)
Supplement: Supplementary Information [file sdata2018293-s1.pdf]

RCSB Protein Data Bank

Supplementary Material: Outlier analyses of the Protein Data Bank archive using a Probability-Density-Ranking approach

Chenghua Shao, Zonghong Liu, Huanwang Yang, Sijian Wang, Stephen K. Burley

Table of Contents

Supplementary Results 2
Impact of different kernel bandwidths and kernel types 2
Comparison of probability density and PDR outliers of different experimental methods 2
Supplementary Tables 4
Table S1: Summary and PDR outlier boundaries of PDB data 4
Table S2: 50%-95% Most Probable Ranges (MPR) of PDB data 5
Table S3: Impact of bandwidth selection on calculating PDR outliers and MPRs 6
Supplementary Figures 7
Figure S1: Distribution and PDR outliers of additional PDB data 7
Figure S1a 8
Figure S1b 9
Figure S1c 10
Figure S1d 11
Figure S1e 12
Figure S1f 13
Figure S1g 14
Figure S1h 15
Figure S1i 16
Figure S1j 17
Figure S1k 18
Figure S1l 19
Figure S2: Comparison of data distribution and outliers from different experimental methods 20
Figure S2a 21
Figure S2b 22
Figure S2c 23
Figure S2d 24
Figure S2e 25
Figure S3: Probability density estimates based on different kernel bandwidth selections 26
Figure S4: Comparison of results from Gaussian and Uniform/Box kernels 27

Supplementary Results

Impact of different kernel bandwidths and kernel types

Probability Density Estimate is closely associated with the size and type of kernels it uses. Supplementary Table S3 and Figure S3 displays outcome and comparison of different bandwidth selections on 10,000 simple random sample of the estimated B factor. A special type of k-Nearest-Neighbor (kNN) kernel was also included in this comparison. Since the distributions from bandwidth between 1.3 and 2.0 were extremely similar, only some representatives from Table S3 were used for Figure S3 for the sake of clarity. For fixed-length bandwidths, greater bandwidth buries local features and thus leads to smoother probability density, with the central peak also lowered because the tail region receives more contribution from data-richer region covered by the broader bandwidth. On the other hand, the diminishing of local features may be undesired if one meant to study local cluster or outliers. The 5% PDR outliers are fairly consistent for all bandwidth selections, whereas the 1% PDB outliers are relatively different at smaller bandwidths due to the presence of local data clusters that are smoothed under greater bandwidth. We concluded that bigger bandwidth and 5% PDR outliers should be used if the goal is to have a crude range and outlier assessment, whereas smaller bandwidth and 1% PDR outliers should be used if one needs to study local distribution features, and the bandwidth from Equation (2) is a good starting bandwidth to use.

The two-step adaptive kernel estimation (h.var in the plot) has the most smoothed distribution, because the local bandwidth is inversely proportional to the density estimate at the location -- the tail region receives much bigger bandwidth whereas the peak region receives smaller bandwidth. The other adaptive kernel, the kNN method, demonstrated more problems: it is sensitive to local features even at the peak, and produces overestimated density at the tail region. The overall estimate by kNN method is also not density function due to the infinite integral. Therefore, we concluded the two-step adaptive kernel and kNN methods are not appropriate for studying local distribution and outliers of PDB data.

We also accessed the impact of other different types of kernels, in addition to kNN. Figure S4 shows the comparison of the probability density estimates and PDR outliers between Uniform (Rectangular or Box) kernel and Gaussian kernel, with either the same or different bandwidths. The results demonstrated that, among all Euclidean distance-based kernels with fixed-length bandwidth, the types of kernels have less significant impact than the size of bandwidth in terms of overall shape of the distribution and PDR outliers. Uniform kernel, due to its non-smooth nature does produce non-smooth density estimates at certain regions, and therefore needs greater bandwidth to have a smoother density estimates (Figure S4b & S4d).

Comparison of probability density and PDR outliers of different experimental methods

As indicated in the conditional data distribution of the results, to have a homogeneous data set for PDR outliers is crucial for its usefulness. Since PDB is an experiment-based archive, the very first factor being considered is the type of experimental method. 18 of the 22 data sets being described here are specific to MX method only. Four data sets (Molecular Weight, Clashscore, Ramachandran Violations, and Rotamer Violations) are pertaining to all three experimental methods: Macromolecular Crystallography (MX), Electron Microscopy (EM), and Nuclear Magnetic Resonance Spectroscopy (NMR). The comparison of method-specific data distributions is illustrated in Figure S2.

Figure S2a shows the overlay of the probability density estimates of Clashscore from all three methods. For MX method, most data are concentrated at the relatively lower Clashscore region, with only 3.2% data beyond the score of 34 that is the 5% PDR outlier boundaries for the data from all three methods (Table S1). Whereas for

both EM and NMR methods there are ~20% data greater than the score of 34. Then the data were separated based on methods, and the Clashscore distributions and PDR outliers for each method were calculated separately and displayed in Figure S2b. The boundaries for EM and NMR methods are much higher than that for MX method.

Figures S2c and S2d display the method-specific distributions and PDR outliers for Ramachandran and Rotamer Violations. Both figures demonstrate different distributions and PDB outliers for different methods. Figure 3 in the results section is a display of Molecular Weight in crystal's asymmetric unit for MX method only, whereas Figure S2e demonstrates the distributions for all methods. Because there is no asymmetric unit for most of EM and NMR structures, all atoms of the modeled sample were added together for EM and NMR structures as their Molecular Weight in comparison to the asymmetric unit Molecular Weight of MX structures. The results show that NMR method was mostly used to study molecules of size below 20 kDa, and common MX research targets could go up to 200 kDa, whereas EM is frequently applied on big molecular complexes such as 2000 kDa target.

Supplementary Tables

Table S1: Summary and PDR outlier boundaries of PDB data

PDB data item	Number of Entries	Parametric fitting				Percentile								Probability Density				
		mean	sd	skewness	kurtosis	median	Q1	Q3	IQR	0.5%	99.5%	2.5%	97.5%	mode	1% PDR Boundary		5% PDR Boundary	
															Low	High	Low	High
Rfree	123849	0.236	0.039	0.174	3.955	0.236	0.21	0.261	0.051	0.133	0.352	0.158	0.314	0.235	0.127	0.344	0.157	0.312
clashscore	141317	10.784	16.323	8.719	214.782	6.31	3.28	12.25	8.97	0	100.564	0.45	49.121	3.1	NA	75.36	NA	34
percent ramachandran violations(%)	137731	0.903	2.333	7.374	106.487	0.18	0	0.76	0.76	0	14.904	0	6.82	0	NA	10.82	NA	4.29
reflection data multiplicity	106032	8.031	104.402	190.324	43933.28	5.1	3.6	7.3	3.7	1.63	41.5	2	20	3.651	NA	28.1	1.3	14.75
molecular weight in asymmetric unit(Da)	124243	98753.36	479589.5	99.797	16570.05	50667.4	30385.3	94788.3	64403	3942.013	1070175	10675.64	379722.5	32818.2	NA	501148	NA	245404
crystal Matthews coefficient(Å^3/Da)	128668	2.671	0.781	21.354	2156.319	2.5	2.21	2.91	0.7	1.67	5.85	1.86	4.52	2.273	1.476	5.32	1.717	4.1
average B factor of protein atoms(Å^2)	111964	38.115	27.168	3.443	31.187	31.062	21.129	46.812	25.683	7.531	170.105	10.678	106.292	22.192	1.89	136.142	5.776	87.105
average B factor of nucleic acid atoms(Å^2)	6933	64.843	47.316	2.633	16.816	53.267	34.598	82.236	47.639	6.878	288.708	11.779	187.626	37.913	NA	238.499	1	150.16
average B factor of ligand atoms(Å^2)	86066	45.971	29.393	2.718	21.531	39.466	26.85	57.158	30.308	7.28	176.67	11.747	119.456	30.54	0.603	153.385	5.69	99.84
average B factor of water atoms(Å^2)	105527	37.594	12.915	3.082	62.991	35.722	29.528	43.53	14.002	11.676	86.82	18.67	66.574	33.007	8.606	81.05	16.359	63.12
B factor estimated from Wilson plot(Å^2 Depositor-reported)	53333	34.792	26.205	5.866	117.799	27.71	18.9	43.2	24.3	5.1	141.229	9	95.153	19.58	NA	118.31	4.123	81.4
B factor estimated from Wilson plot(Å^2 PDB-calculated)	116209	33.923	26.48	5.509	79.556	27.149	18.53	41.354	22.824	6.54	153.806	9.24	95.018	19.383	1.397	125.246	5.001	78.387
crystal solvent percentage(%)	128714	51.363	10.105	0.172	3.601	50.51	44.39	57.72	13.33	25.306	79	33.67	72.78	49.42	27.6	80.4	33.2	72.3
crystal mosaicity	2660	0.489	0.619	16.837	491.398	0.376	0.17	0.67	0.5	0.04	2.169	0.05	1.517	0.149	NA	1.853	NA	1.267
Rfree minus Rwork	122982	0.041	0.017	0.787	5.28	0.039	0.029	0.051	0.022	0.005	0.1	0.013	0.08	0.037	0.001	0.094	0.01	0.076
reflection high resolution limit(Å)	130858	2.202	1.156	25.495	1161.253	2.07	1.8	2.5	0.7	1	5.8	1.2	3.5	1.978	0.8	4.1	1.084	3.3
reflection data indexing chi-square	11822	1.285	1.303	33.78	1627.396	1.046	0.982	1.301	0.319	0.52	5.207	0.765	2.844	1.007	0.306	3.764	0.597	2.446
reflection data Intensity/Sigma	103377	18.85	245.217	166.713	30993.2	14.1	9.89	20.3	10.41	2.3	63.1	4.6	43.9	11.008	0.097	54.245	2.25	37.4
reflection data Rmerge	88441	0.096	0.342	74.438	8457.843	0.077	0.059	0.101	0.042	0.021	0.44	0.034	0.207	0.061	0.003	0.287	0.024	0.173
reflection data completeness(%)	122049	96.37	7.004	-6.938	78.235	98.6	95.7	99.7	4	61.124	100	80.8	100	99.78	74.5	NA	86.48	NA
percent rotamer violations(%)	137522	4.834	6.452	3.224	17.618	2.68	1.12	5.88	4.76	0	38.844	0	24.65	0.92	NA	33.08	NA	17.19
percent RSRZ violations(%)	113450	3.976	4.196	5.377	82.073	2.93	1.34	5.4	4.06	0	21.05	0	13.66	1.16	NA	17.46	NA	11.04

Table S2: 50%-95% Most Probable Ranges (MPR) of PDB data

PDB data item	50%MPR	60%MPR	70%MPR	80%MPR	90%MPR	95%MPR
Rfree	0.21--<br>0.261	0.204--<br>0.268	0.196--<br>0.275	0.188--<br>0.285	0.173--<br>0.299	0.157--<br>0.312
clashscore	1.01--<br>7.11	0.56--<br>8.69	0.14--<br>11.07	0--<br>14.49	0--<br>22.75	0--<br>34
percent ramachandran violations(%)	0--<br>0.15	0--<br>0.34	0--<br>0.57	0--<br>1.03	0--<br>2.32	0--<br>4.29
reflection data multiplicity	2.758--<br>5.05	2.69--<br>5.5	2.5--<br>7.442	1.65--<br>8.3	1.472--<br>11.55	1.3--<br>14.75
molecular weight in asymmetric unit(Da)	13396.9--<br>55743.2	10517.1--<br>67565.6	7560.54--<br>85235.2	4979.67--<br>111249	649.76--<br>168925	468.55--<br>245404
crystal Matthews coefficient(Å^3/Da)	2.036--<br>2.63	2--<br>2.77	1.947--<br>2.91	1.874--<br>3.15	1.787--<br>3.61	1.717--<br>4.1
average B factor of protein atoms(Å^2)	13.761--<br>34.708	12.417--<br>39.232	11.071--<br>45.093	9.582--<br>53.508	7.493--<br>69.261	5.776--<br>87.105
average B factor of nucleic acid atoms(Å^2)	22.445--<br>62.349	18.213--<br>69.934	14.821--<br>81.117	11.302--<br>95.812	5.058--<br>119.21	1--<br>150.16
average B factor of ligand atoms(Å^2)	19.029--<br>45.975	16.75--<br>50.74	14.357--<br>56.94	11.845--<br>65.87	8.405--<br>81.915	5.69--<br>99.84
average B factor of water atoms(Å^2)	27.21--<br>40.625	25.737--<br>42.744	24.086--<br>45.515	22.218--<br>49.378	19.175--<br>55.94	16.359--<br>63.12
B factor estimated from Wilson plot(Å^2 Depositor-reported)	12.45--<br>31.3	11.064--<br>35.77	9.52--<br>41.55	7.892--<br>50.76	5.9--<br>66.61	4.123--<br>81.4
B factor estimated from Wilson plot(Å^2 PDB-calculated)	12.254--<br>30.513	10.877--<br>34.295	9.462--<br>39.519	8.116--<br>47.66	6.334--<br>62.421	5.001--<br>78.387
crystal solvent percentage(%)	42.62--<br>55.63	41.18--<br>57.41	39.83--<br>59.88	38.18--<br>63.22	35.89--<br>68.58	33.2--<br>72.3
crystal mosaicity	0.04--<br>0.387	0.03--<br>0.482	0.03--<br>0.6	0.03--<br>0.739	0.03--<br>0.989	0.03--<br>1.267
Rfree minus Rwork	0.027--<br>0.048	0.024--<br>0.051	0.021--<br>0.055	0.018--<br>0.059	0.014--<br>0.067	0.01--<br>0.076
reflection high resolution limit(Å)	1.656--<br>2.33	1.585--<br>2.421	1.484--<br>2.592	1.447--<br>2.8	1.26--<br>3.075	1.084--<br>3.3
reflection data indexing chi-square	0.922--<br>1.097	0.883--<br>1.156	0.842--<br>1.297	0.788--<br>1.536	0.723--<br>1.98	0.597--<br>2.446
reflection data Intensity/Sigma	7.2--<br>16.28	6.36--<br>17.9	5.59--<br>20.18	4.64--<br>23.6	3.4--<br>30.2	2.25--<br>37.4
reflection data Rmerge	0.048--<br>0.088	0.045--<br>0.094	0.04--<br>0.103	0.036--<br>0.116	0.03--<br>0.141	0.024--<br>0.173
reflection data completeness(%)	98.6--<br>100	97.8--<br>100	96.5--<br>100	94.5--<br>100	90.8--<br>100	86.48--<br>100
percent rotamer violations(%)	0--<br>2.68	0--<br>3.65	0--<br>5	0--<br>7.03	0--<br>11.22	0--<br>17.19
percent RSRZ violations(%)	0.05--<br>3.33	0--<br>3.74	0--<br>4.76	0--<br>6.16	0--<br>8.59	0--<br>11.04

Table S3: Impact of bandwidth selection on calculating PDR outliers and MPRs

Name	Bandwidth	1% PDR outliers left bound	1% PDR outliers right bound	5% PDR outliers left bound	5% PDR outliers right bound	50% MPR width
h.iqr	2.8	NA	117.652	3.655	77.068	17.999
h.amise	9.767	NA	121.531	NA	76.661	18.456
h.bcv	1.726	NA	112.135	4.976	77.388	17.941
h.ccv	1.564	NA	112.135	5.063	77.464	17.945
h.mcv	1.968	NA	112.942	4.562	77.217	17.94
h.mlcv	4.994	NA	121.531	NA	76.661	17.876
h.tcv	1.574	NA	112.135	5.063	77.464	17.945
h.ucv	1.365	1.859	112.135	5.188	77.464	17.947
h.knn		NA	105.308	NA	76.661	17.924
h.var		NA	121.531	NA	76.661	18.328

Different kernel bandwidths are applied to the same data set of 10000 sample of B factor values from Wilson Plot, in the unit of $Å^2$. h.knn and h.var are variable-length and the rest are fixed-length bandwidth with size indicated in the 2nd column. Each bandwidth is named by letter h, a dot, followed by the abbreviation of the method: h.iqr based on IQR as indicated in Equation (2); h.amise, based on Asymptotic Mean Integrated Squared Error; h.bcv, based on Biased Cross-Validation; h.ccv, based on Complete Cross-Validation; h.mcv, based on Modified Cross-Validation; h.mlcv, based on Maximum-Likelihood Cross-Validation; h.tcv, Trimmed Cross-Validation; h.ucv, Unbiased (Least-Squares) Cross-Validation; h.var, Variable kernel density estimator; h.knn, k-Nearest Neighbor used in Equation (3). The left/right bound is decided in the following way: starting from mode and move to lower (left) tail or upper (right) tail, the 1st observation with estimated probability density lower than threshold at the lower tail is the left bound, and 1st at the upper tail is the right bound. "NA" indicates there is no outlier at the specified end for the threshold.

Supplementary Figures

Figure S1: Distribution and PDR outliers of additional PDB data

Distribution of the following additional PDB data sets: (a) B factor estimated from Wilson Plot (Å^2, PDB-calculated); (b) B factor estimated from Wilson Plot (Å^2, Depositor-reported); (c) Crystal solvent percent (%); (d) Crystal mosaicity; (e) Rfree minus Rwork; (f)Reflection high resolution limit (Å); (g)Reflection data indexing Chi-square; (h) Reflection data Intensity/Sigma; (i) Reflection data Rmerge; (j) Reflection data completeness (%); (k) Percent Rotamer violations(%); (l) Percent RSRZ violations (%). Each graph contains three panels showing 5% PDR outliers (upper left), 1% PDR outliers (upper right), and Normal Q-Q plot (bottom left). Figure title indicates the unit of the measurement if applicable. PDR outlier regions are colored in red and non-outlier regions in blue.

Figure S1a

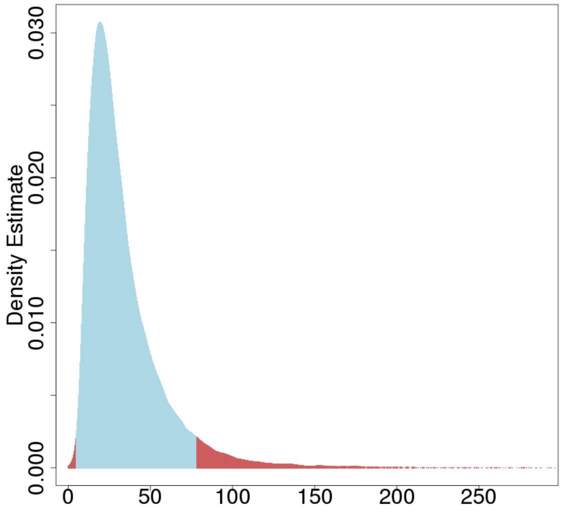
Density Estimate
0.000
0.010
0.020
0.030
0
50
100
150
200
250

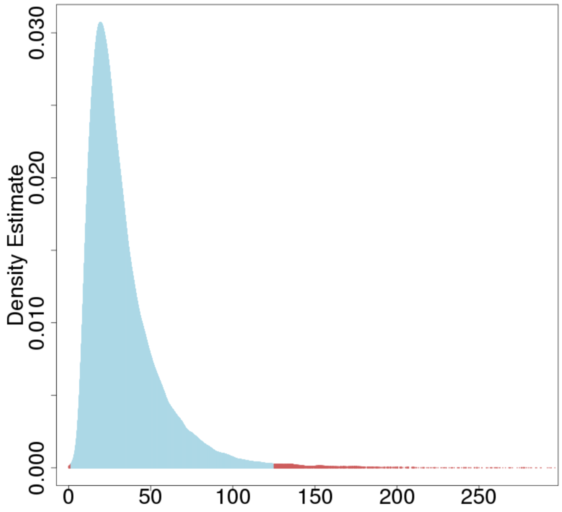
Density Estimate
0.000
0.010
0.020
0.030
0
50
100
150
200
250

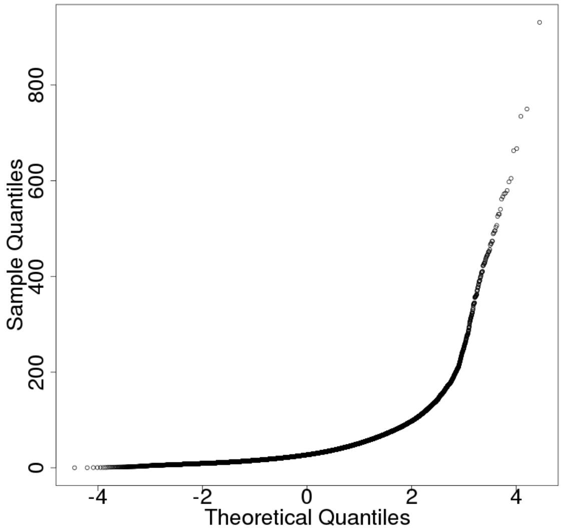
Sample Quantiles
0
200
400
600
800
-4
-2
0
2
4
Theoretical Quantiles

Figure S1b

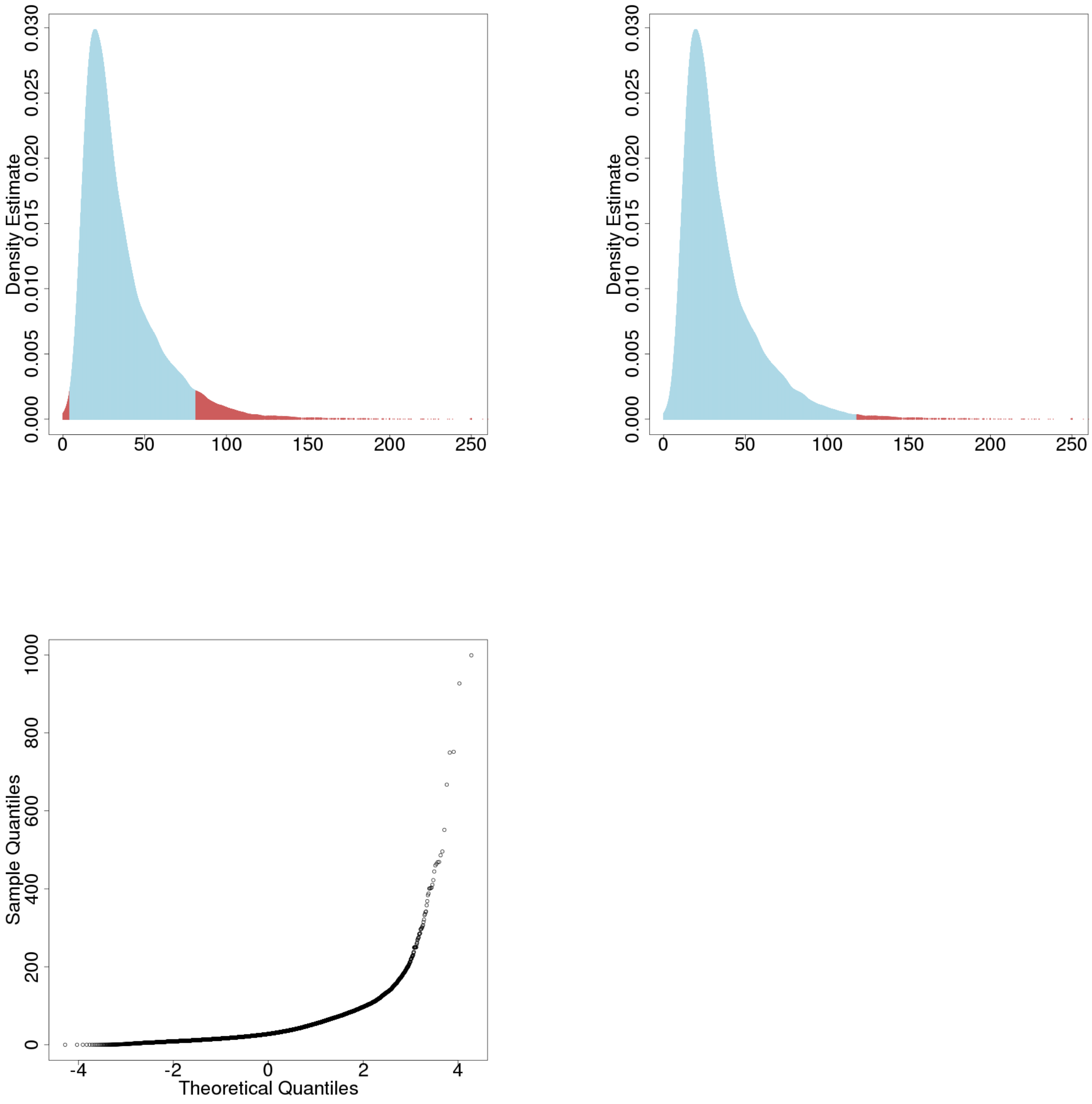

Density Estimate
0.000
0.005
0.010
0.015
0.020
0.025
0.030
0
50
100
150
200
250
Density Estimate
0.000
0.005
0.010
0.015
0.020
0.025
0.030
0
50
100
150
200
250
Sample Quantiles
0
200
400
600
800
1000
-4
-2
0
2
4
Theoretical Quantiles

Figure S1c

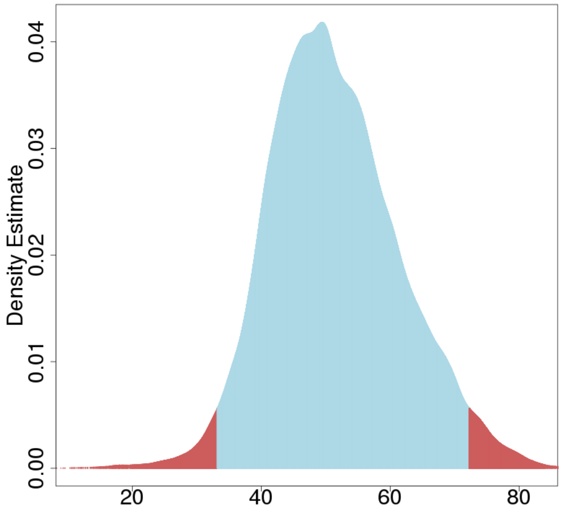

Density Estimate
0.00
0.01
0.02
0.03
0.04
20
40
60
80

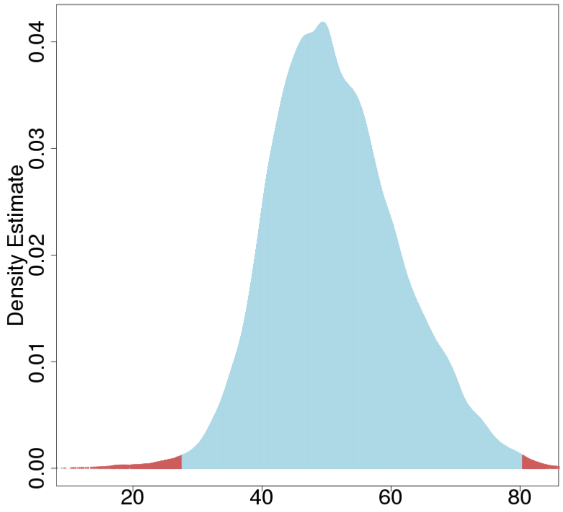

Density Estimate
0.00
0.01
0.02
0.03
0.04
20
40
60
80

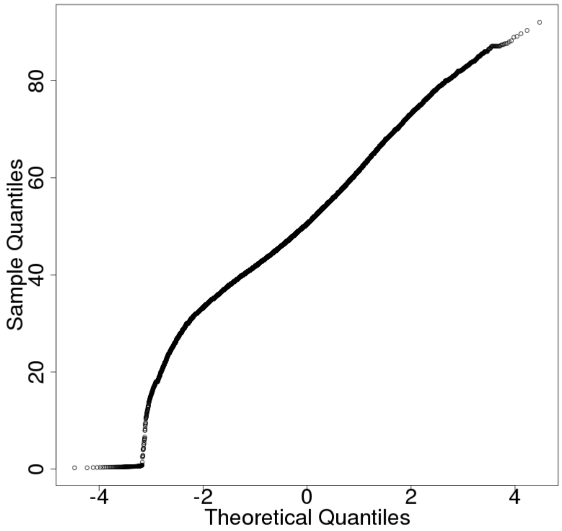

Sample Quantiles
0
20
40
60
80
-4
-2
0
2
4
Theoretical Quantiles

Figure S1d

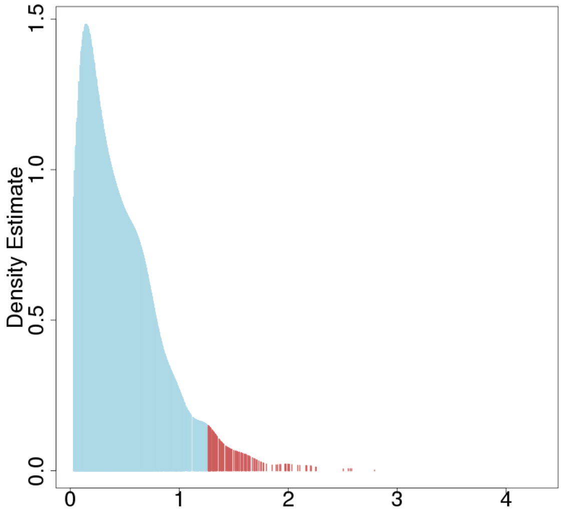

Density Estimate
0.0
0.5
1.0
1.5
0
1
2
3
4

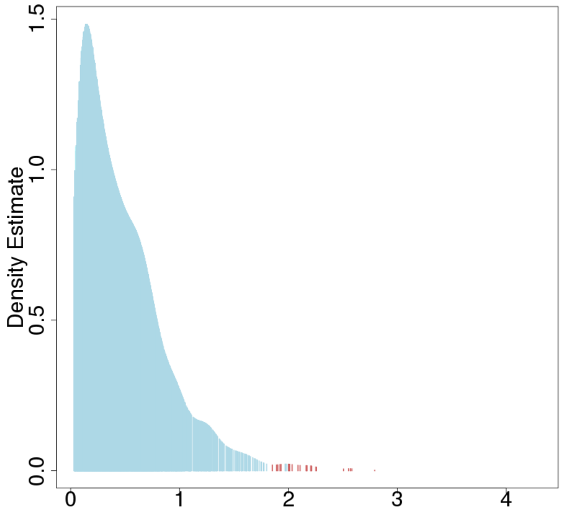

Density Estimate
0.0
0.5
1.0
1.5
0
1
2
3
4

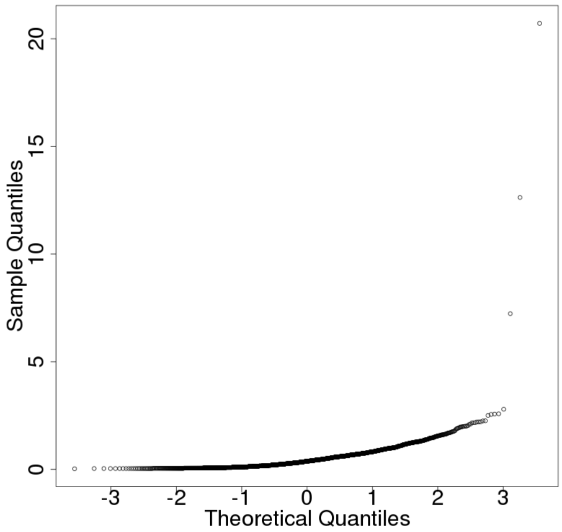

Sample Quantiles
0
5
10
15
20
-3
-2
-1
0
1
2
3
Theoretical Quantiles

Figure S1e

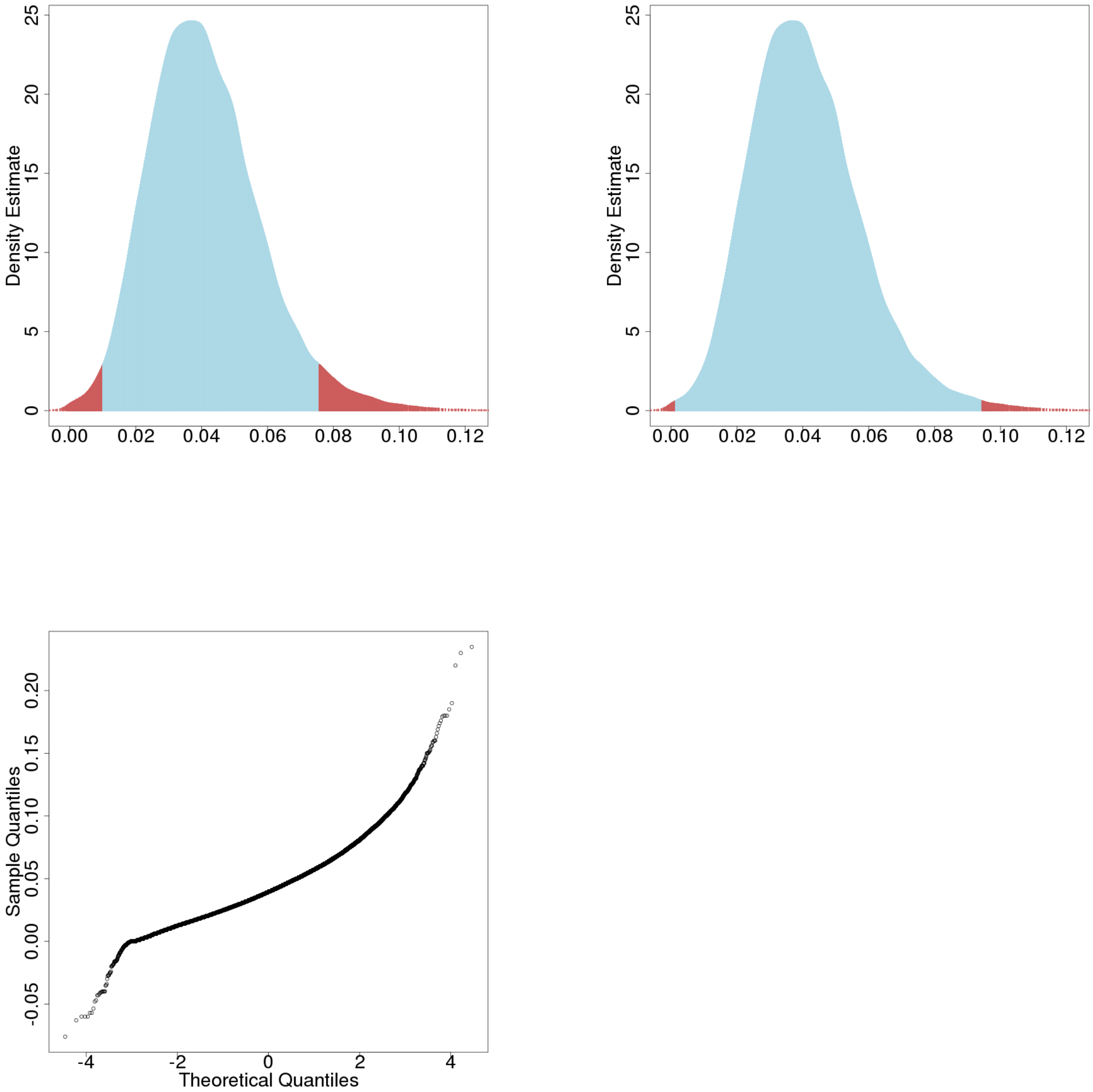

Density Estimate
0
5
10
15
20
25
0.00
0.02
0.04
0.06
0.08
0.10
0.12
Density Estimate
0
5
10
15
20
25
0.00
0.02
0.04
0.06
0.08
0.10
0.12
Sample Quantiles
-0.05
0.00
0.05
0.10
0.15
0.20
-4
-2
0
2
4
Theoretical Quantiles

Figure S1f

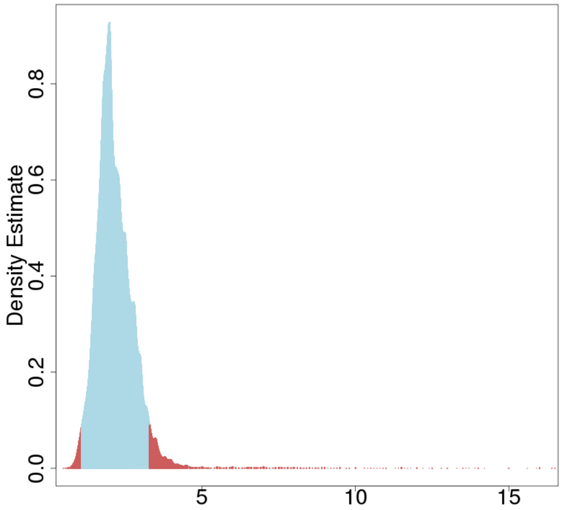
Density Estimate
0.0
0.2
0.4
0.6
0.8
5
10
15

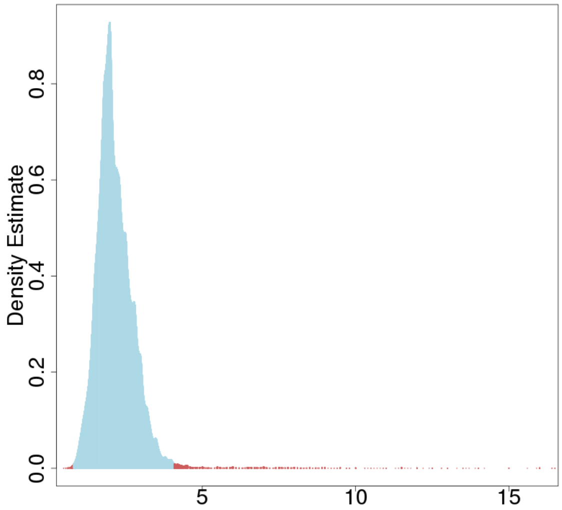
Density Estimate
0.0
0.2
0.4
0.6
0.8
5
10
15

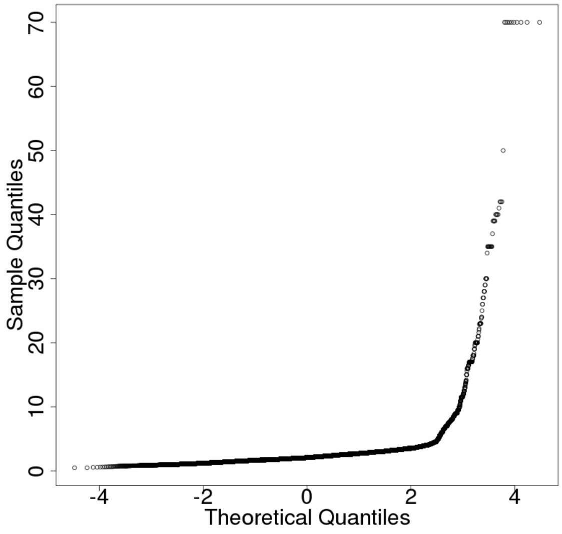
Sample Quantiles
0
10
20
30
40
50
60
70
-4
-2
0
2
4
Theoretical Quantiles

Figure S1g

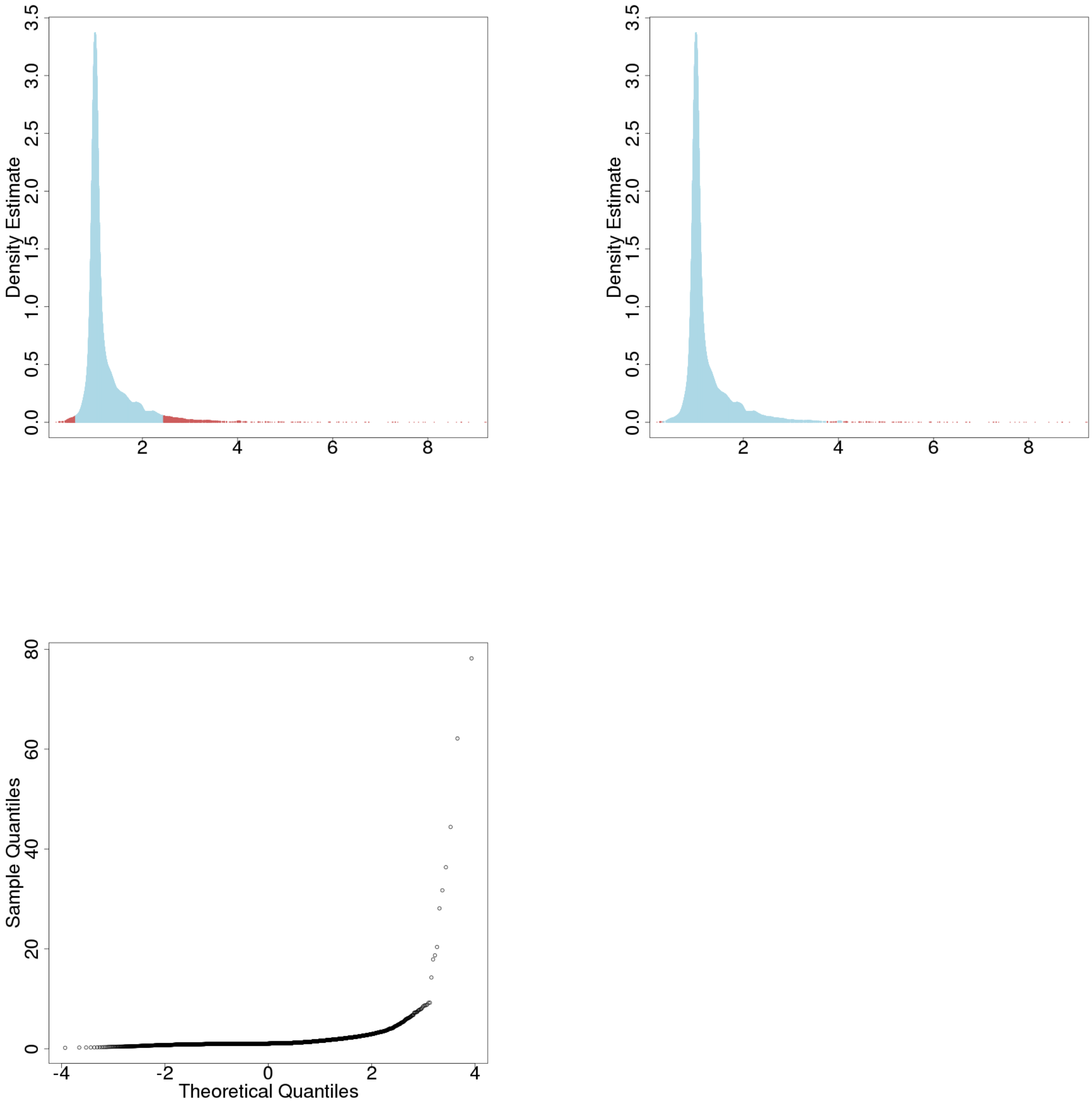
Density Estimate
0.0
0.5
1.0
1.5
2.0
2.5
3.0
3.5
2
4
6
8
Density Estimate
0.0
0.5
1.0
1.5
2.0
2.5
3.0
3.5
2
4
6
8
Sample Quantiles
0
20
40
60
80
-4
-2
0
2
4
Theoretical Quantiles

Figure S1h

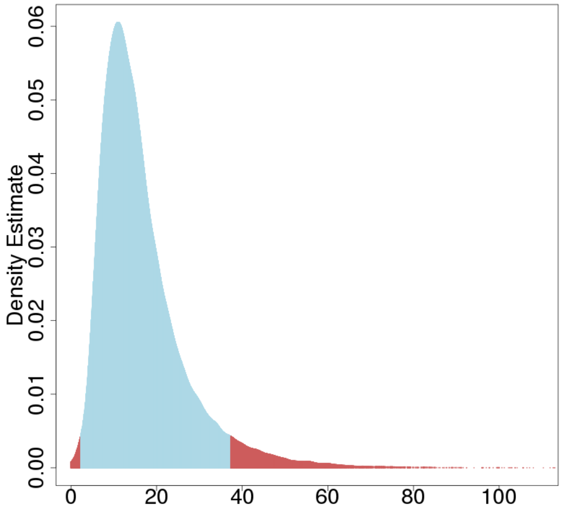

Density Estimate
0.00
0.01
0.02
0.03
0.04
0.05
0.06
0
20
40
60
80
100

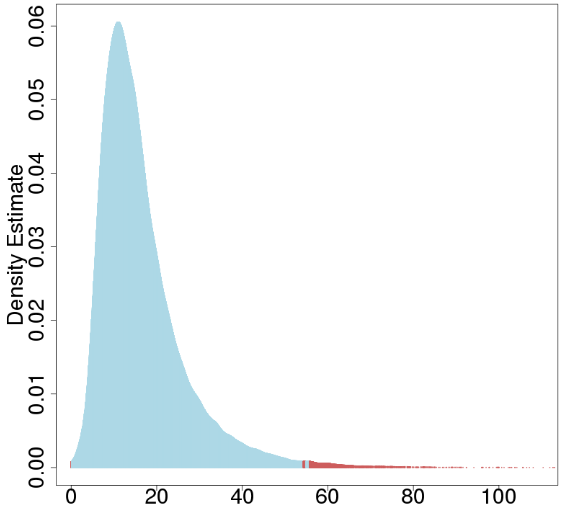

Density Estimate
0.00
0.01
0.02
0.03
0.04
0.05
0.06
0
20
40
60
80
100

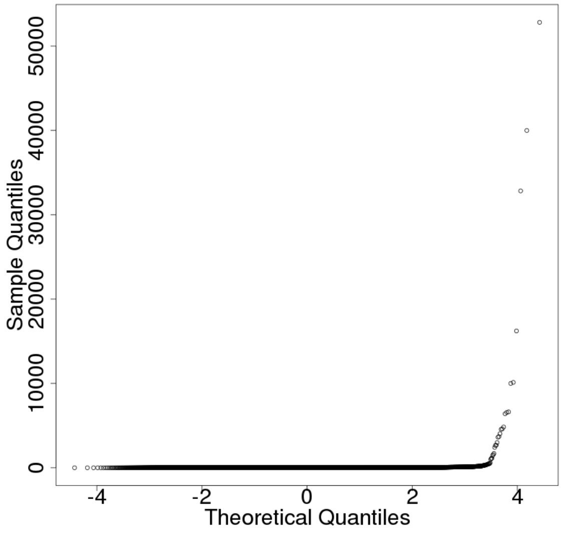

Sample Quantiles
0
10000
20000
30000
40000
50000
-4
-2
0
2
4
Theoretical Quantiles

Figure S1i

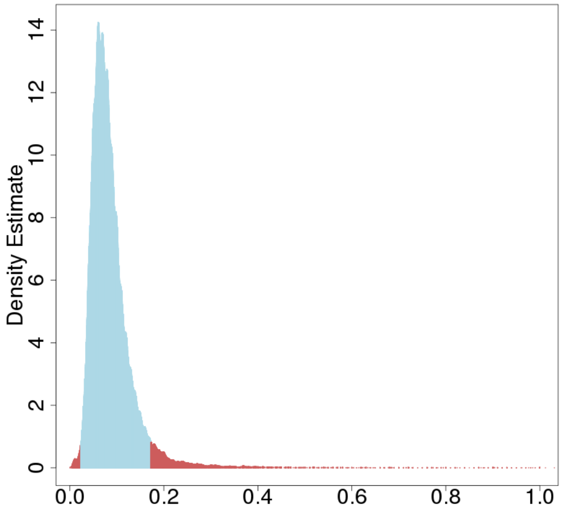
Density Estimate
0 2 4 6 8 10 12 14
0.0 0.2 0.4 0.6 0.8 1.0

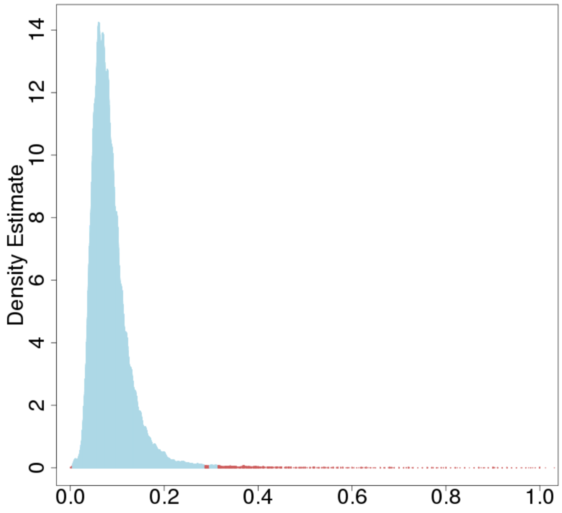
Density Estimate
0 2 4 6 8 10 12 14
0.0 0.2 0.4 0.6 0.8 1.0

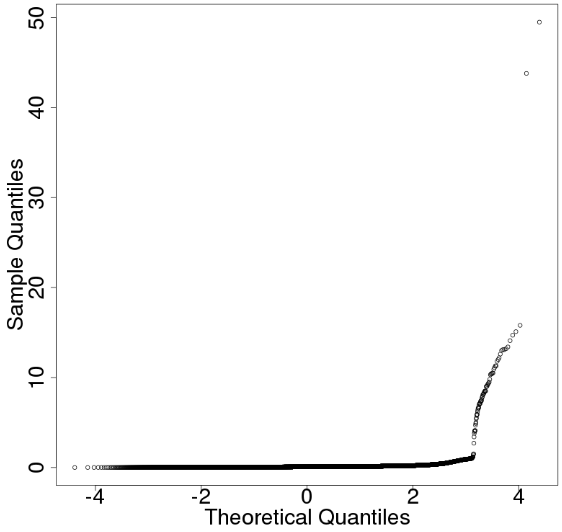
Sample Quantiles
0 10 20 30 40 50
-4 -2 0 2 4
Theoretical Quantiles

Figure S1j

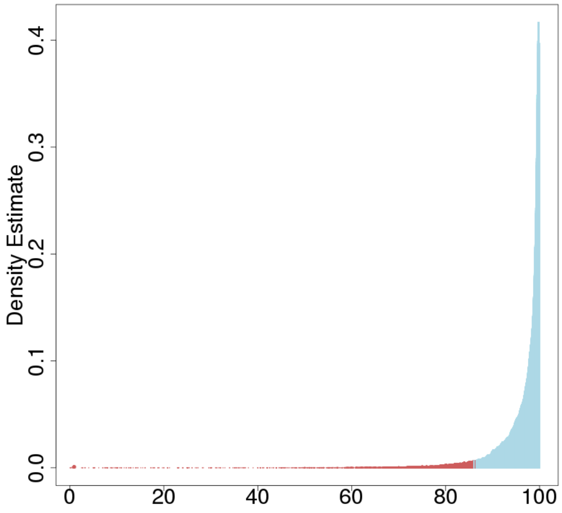

Density Estimate
0.0
0.1
0.2
0.3
0.4
0
20
40
60
80
100

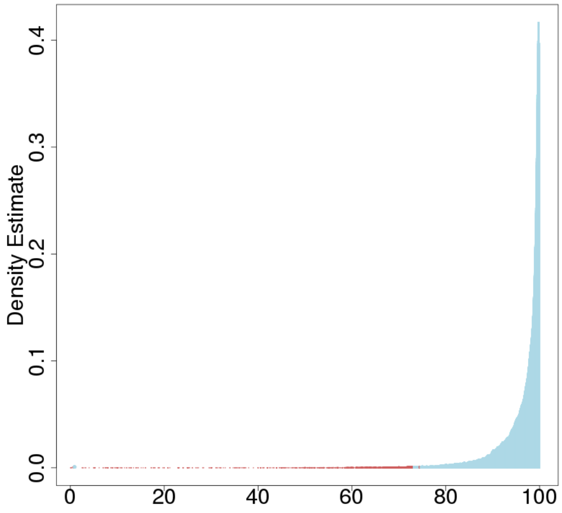

Density Estimate
0.0
0.1
0.2
0.3
0.4
0
20
40
60
80
100

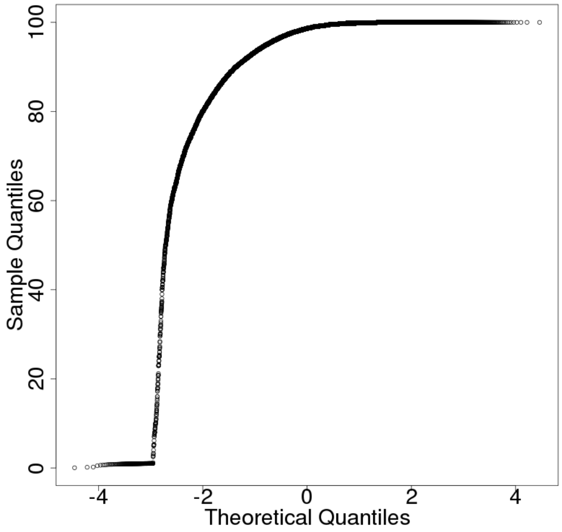

Sample Quantiles
0
20
40
60
80
100
-4
-2
0
2
4
Theoretical Quantiles

Figure S1k

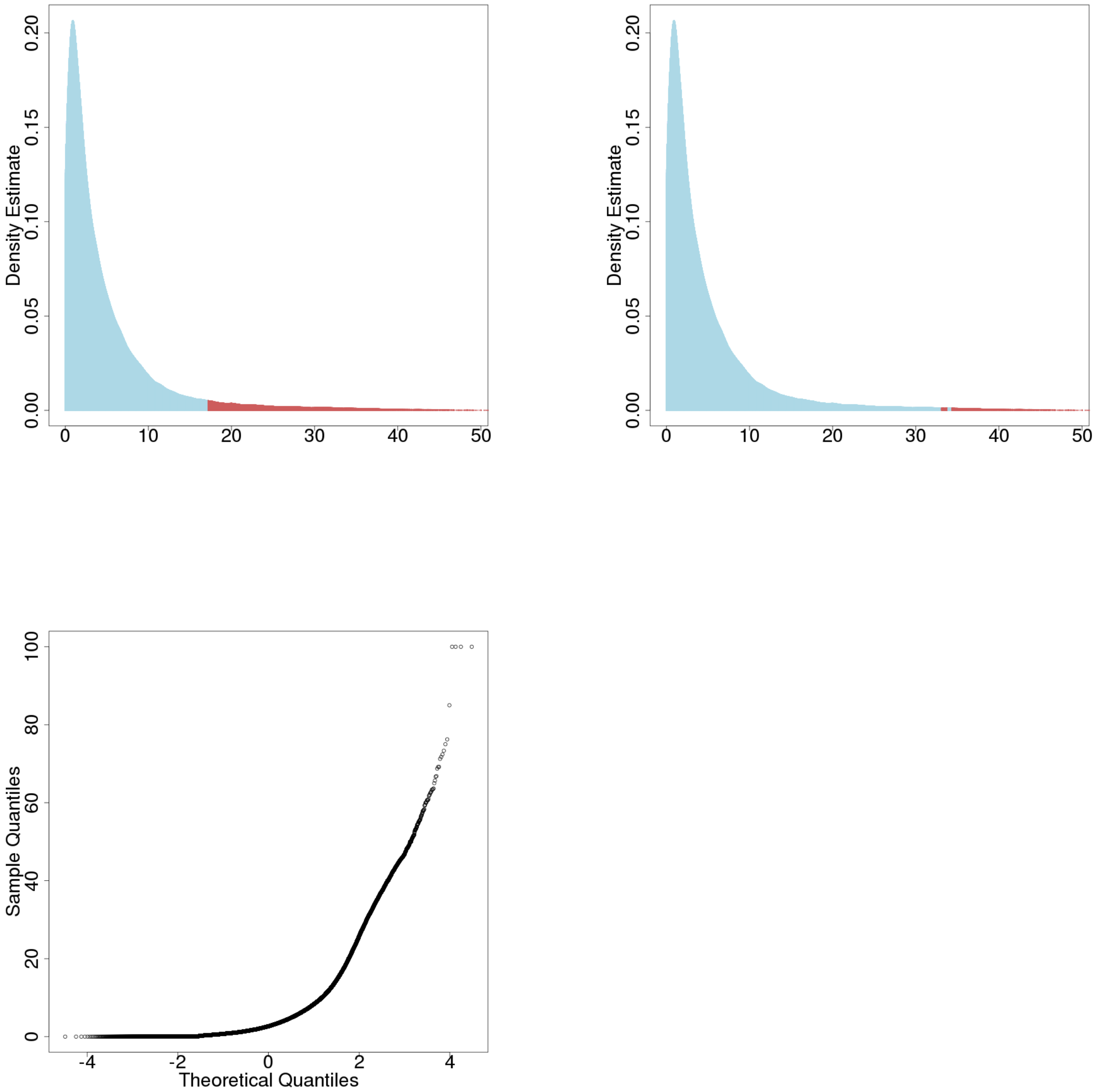

Density Estimate
0.00
0.05
0.10
0.15
0.20
0
10
20
30
40
50
Density Estimate
0.00
0.05
0.10
0.15
0.20
0
10
20
30
40
50
Sample Quantiles
0
20
40
60
80
100
-4
-2
0
2
4
Theoretical Quantiles

Figure S1l

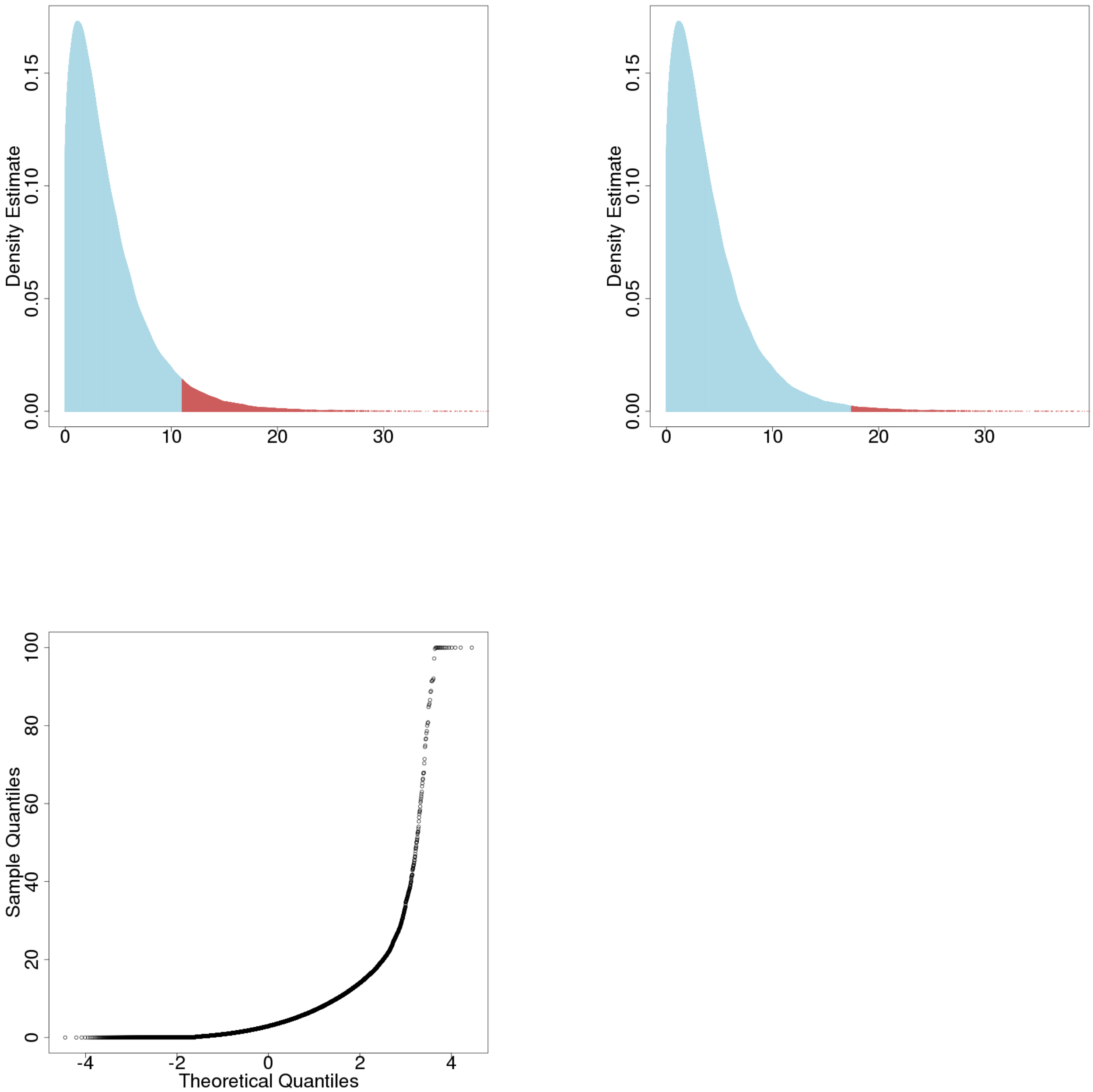
Density Estimate
0.00
0.05
0.10
0.15
0
10
20
30
Density Estimate
0.00
0.05
0.10
0.15
0
10
20
30
Sample Quantiles
0
20
40
60
80
100
-4
-2
0
2
4
Theoretical Quantiles

Figure S2: Comparison of data distribution and outliers from different experimental methods

(a) Overlay of Clashscore data from three experimental methods: Macromolecular Crystallography (MX), Electron Microscopy (EM), and Nuclear Magnetic Resonance Spectroscopy (NMR). (b-e) Method-specific distribution of Clashscore, Ramachandran violations (%), Rotamer violations (%) and Molecular Weight (Da), respectively, with data from each method plotted in separate panels. Figure title indicates the unit of measurement if applicable. PDR outlier region is colored in red and non-outlier region in blue. Because the data range for different method can be very different, each panel in figures b-e displays data at different range, and overlay is only made for Clashscore. Data from hybrid methods were not included.

Figure S2a

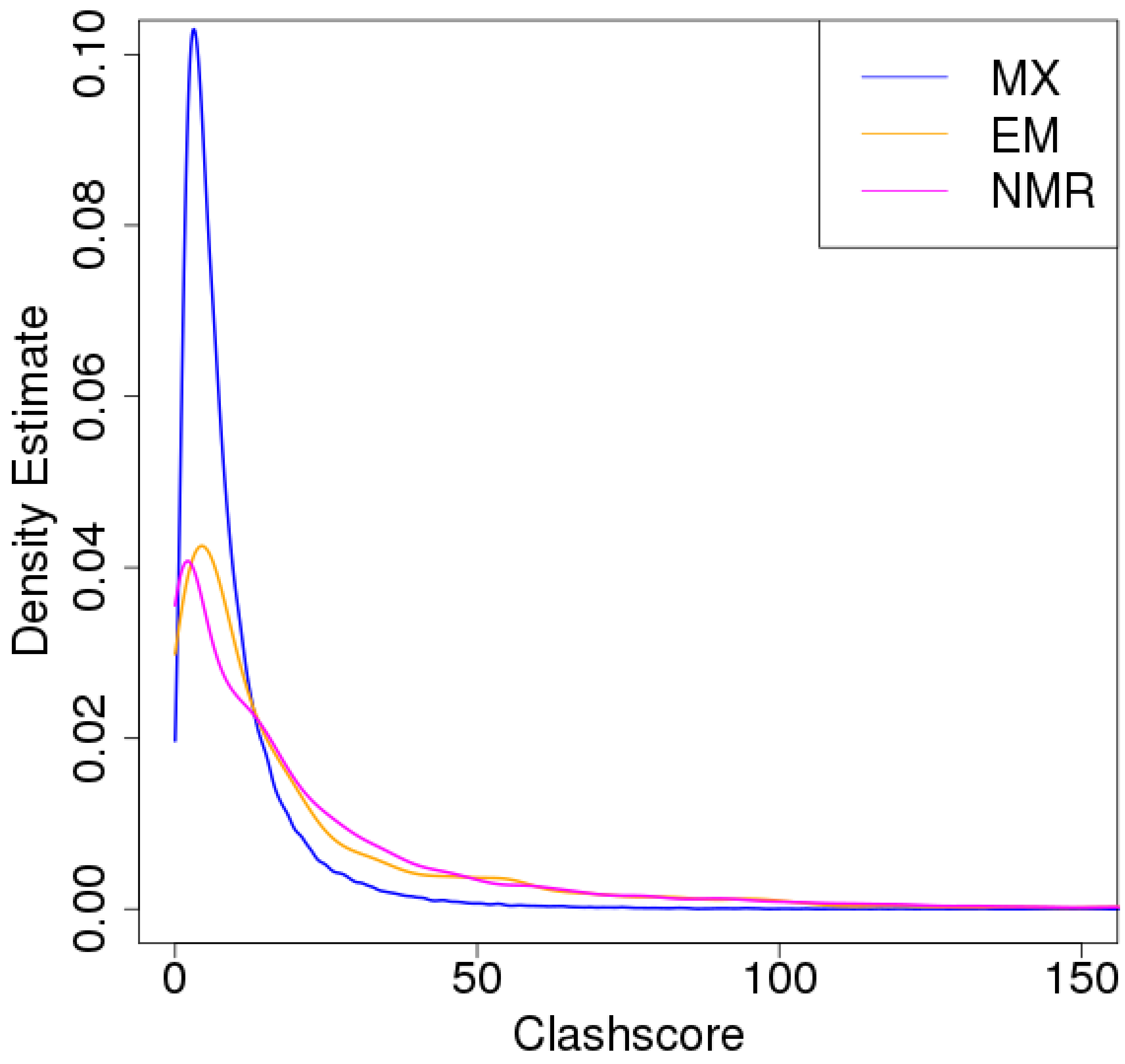
MX
EM
NMR
Density Estimate
0.00
0.02
0.04
0.06
0.08
0.10
0
50
100
150
Clashscore

Figure S2b

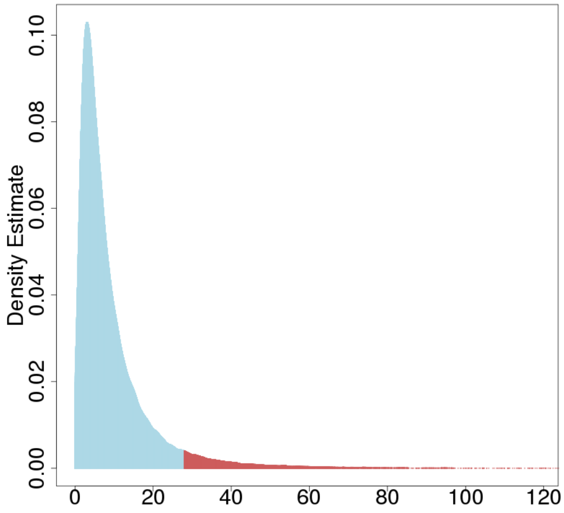

MX

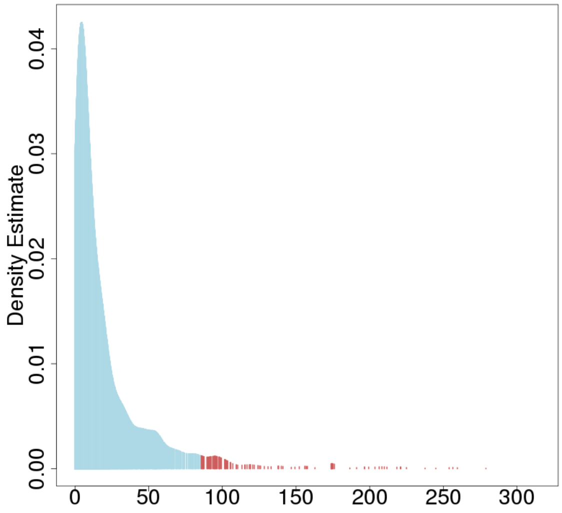

EM

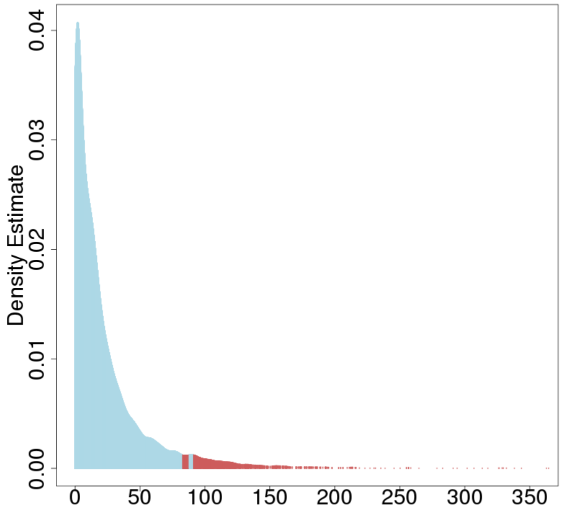

NMR

Figure S2c

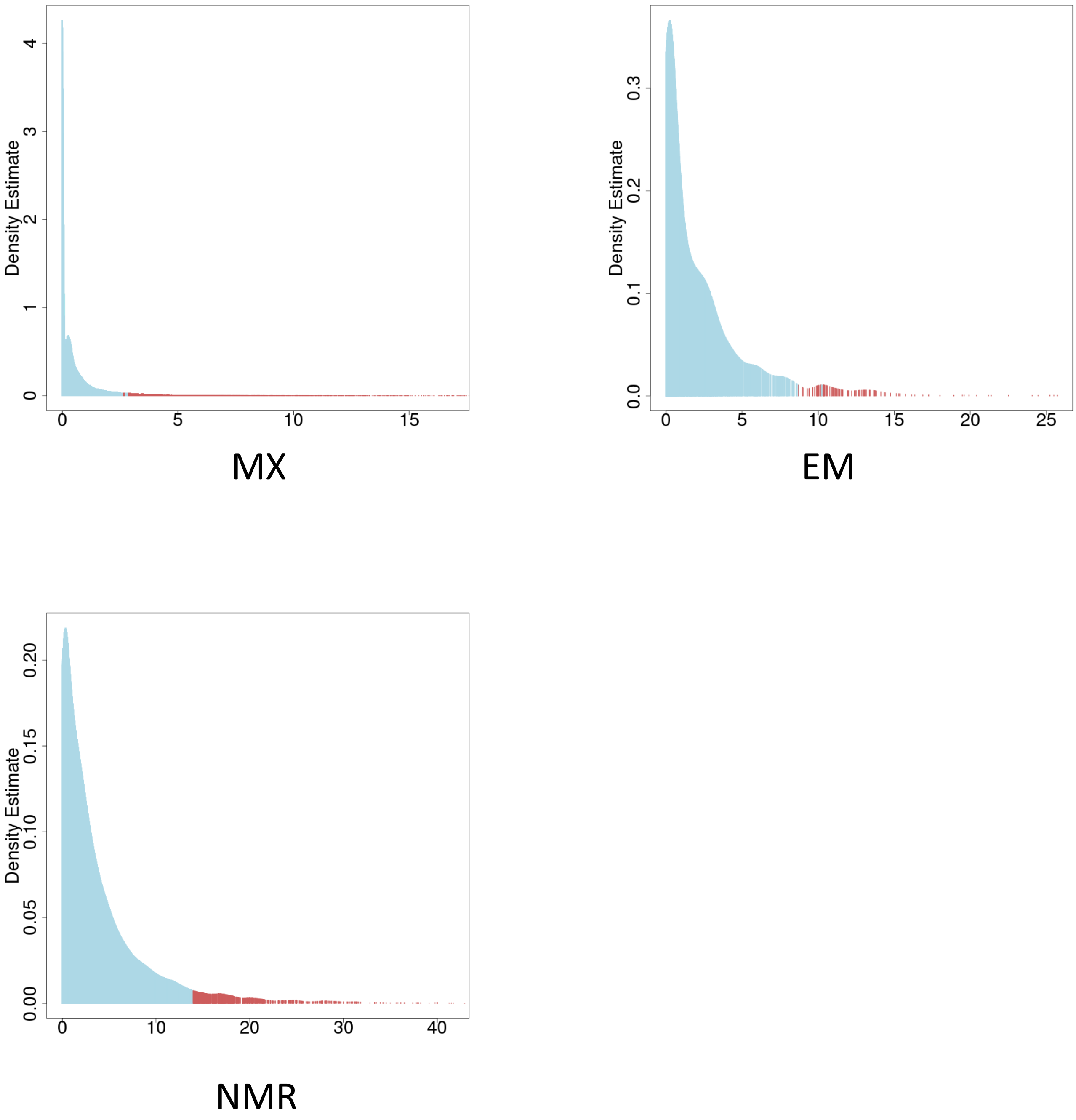
Density Estimate
0
1
2
3
4
0
5
10
15
MX
Density Estimate
0.0
0.1
0.2
0.3
0
5
10
15
20
25
EM
Density Estimate
0.00
0.05
0.10
0.15
0.20
0
10
20
30
40
NMR

Figure S2d

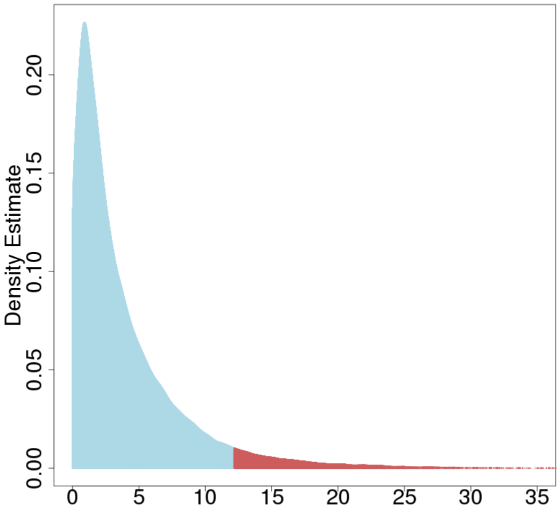

MX

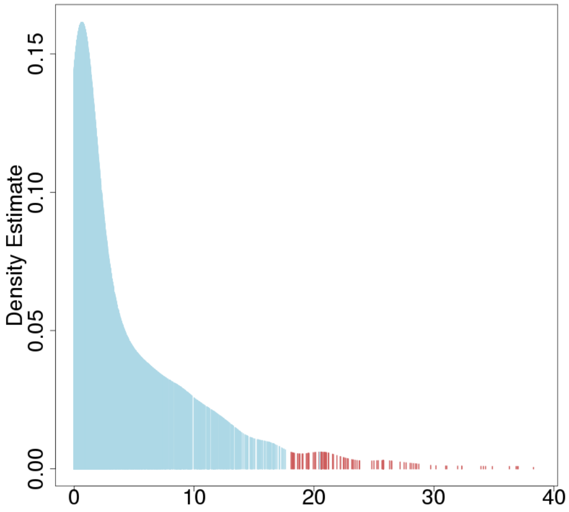

EM

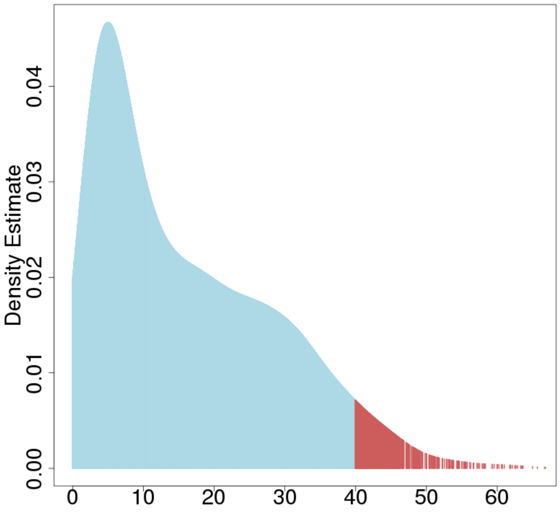

NMR

Figure S2e

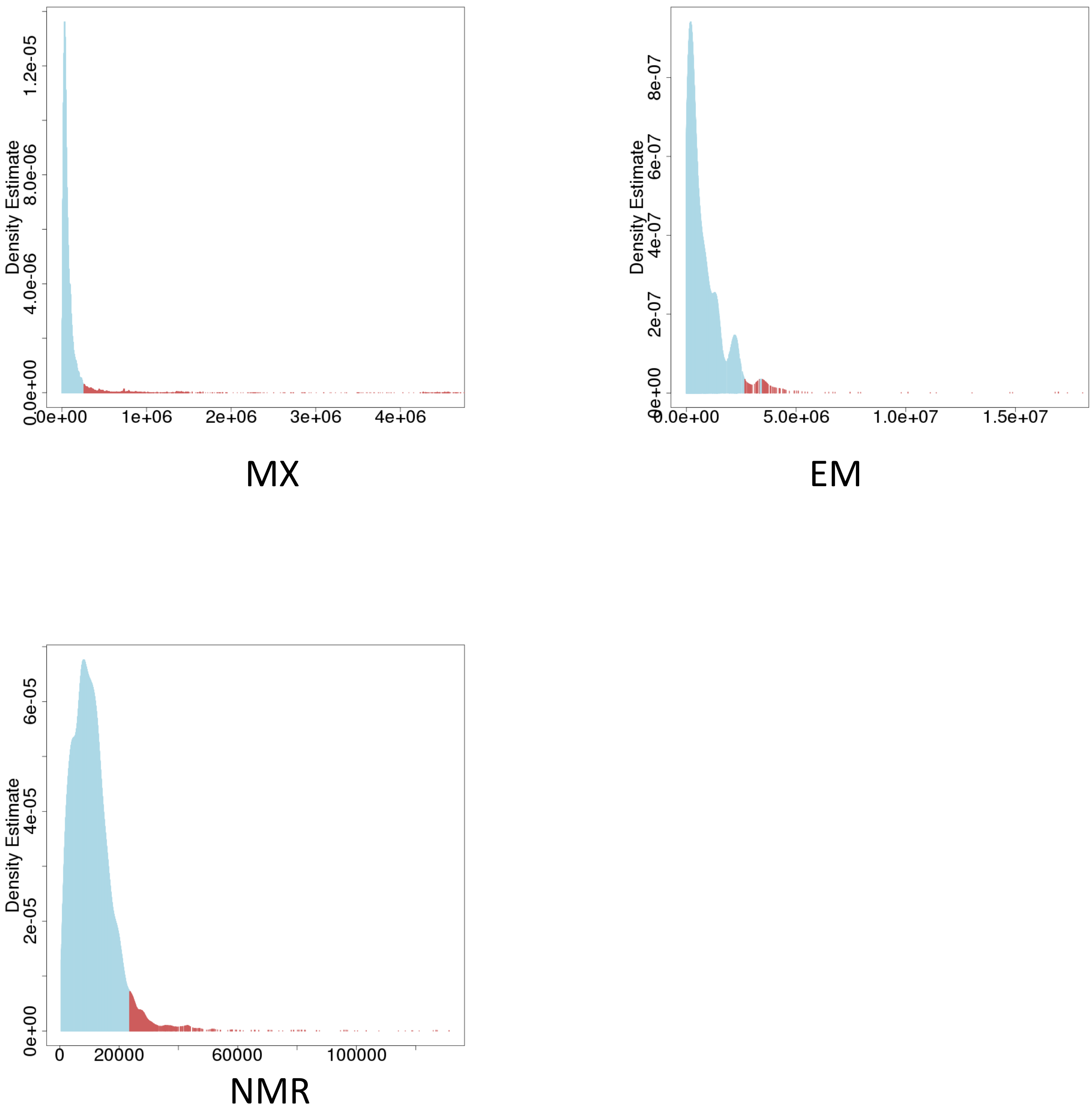
Density Estimate
0.0e+00
4.0e-06
8.0e-06
1.2e-05
0e+00
1e+06
2e+06
3e+06
4e+06
MX
Density Estimate
0e+00
2e-07
4e-07
6e-07
8e-07
0.0e+00
5.0e+06
1.0e+07
1.5e+07
EM
Density Estimate
0e+00
2e-05
4e-05
6e-05
0
20000
60000
100000
NMR

Figure S3: Probability density estimates based on different kernel bandwidth selections

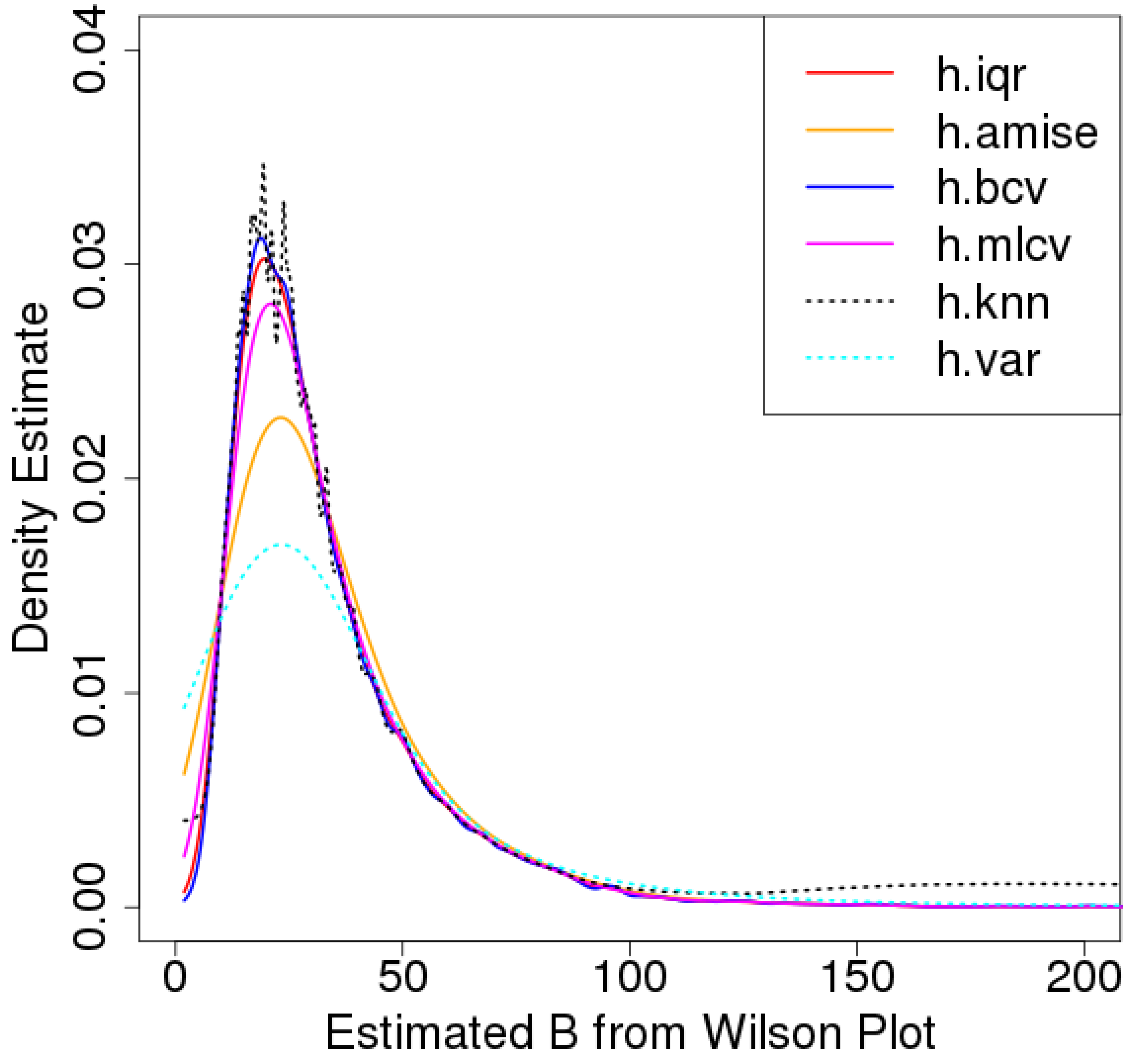

Data being displayed is the estimated isotropic B factor based on Wilson plot. Gaussian kernel is used by default with different bandwidths as indicated, except for kNN kernel that was based on Eq 3. Calculation was conducted on a sample of 10000 PDB X-ray entries from the archive. Solid colored lines for estimation from fixed-length kernel bandwidths and dotted lines from adaptive kernel bandwidths. Legend of Table S3 specifies methods to calculate each bandwidth.

Figure S4: Comparison of results from Gaussian and Uniform/Box kernels

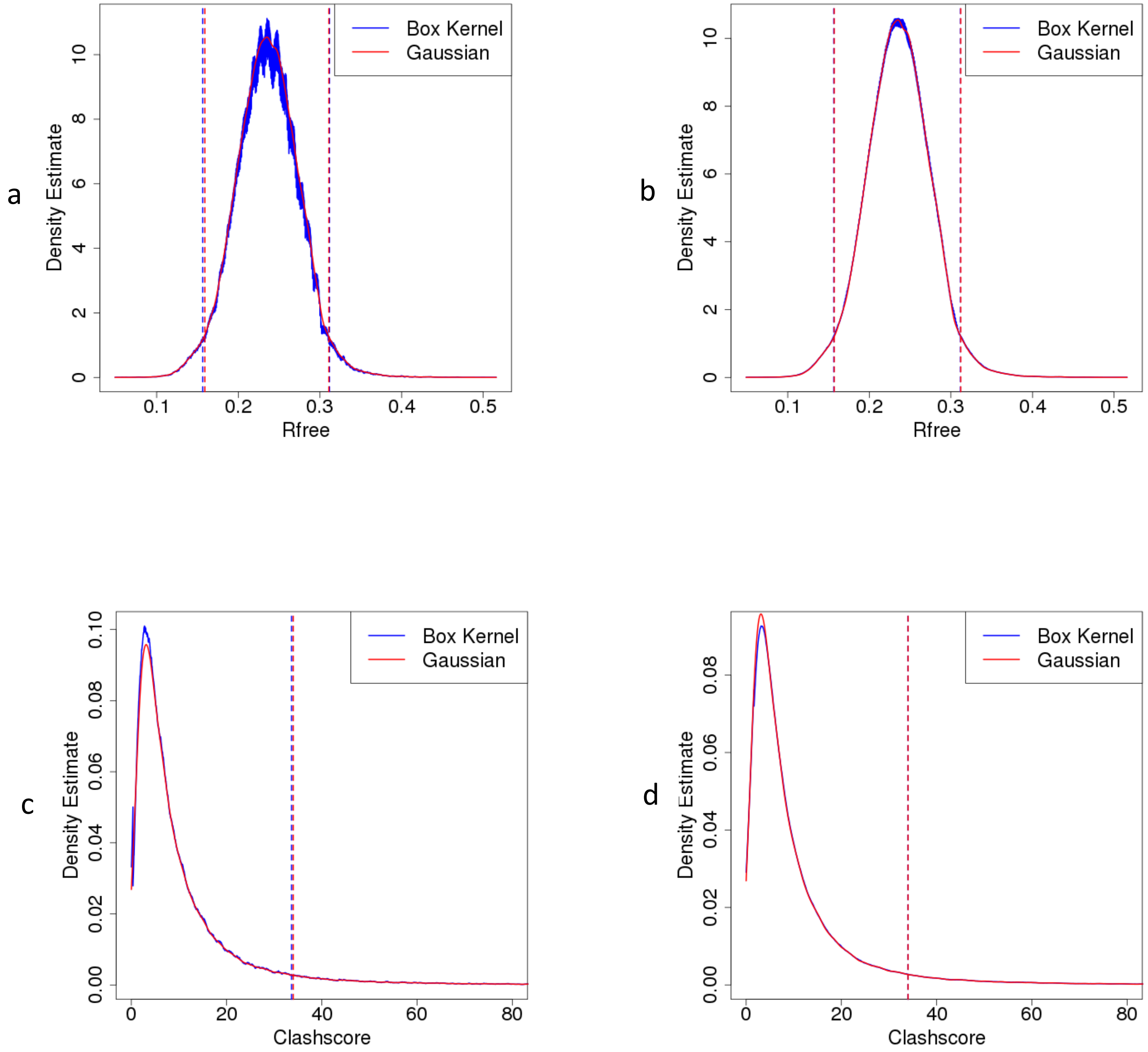

(a & b) Rfree and (c & d) Clashscore distribution overlay of probability density estimated by Uniform/Box kernel (blue) and Gaussian kernel (red). PDR outlier boundaries are also indicated by vertical dashed lines by Uniform kernel (blue) and Gaussian kernel (red). For all panels, Gaussian kernel estimates used bandwidths of h_{opt} based on Eq 2. Uniform kernel estimates used bandwidths of either h_{opt} (a & c) or $5\times h_{opt}$ (b & d). The high-level consistency makes it difficult to see lines of both colors at some regions of the distribution curves or at the outlier boundaries.
